# Engineering Hydrogels for Intrauterine Adhesion Therapy and Endometrial Regeneration

**DOI:** 10.3390/gels12090811

**Published:** 2026-09-04

**Authors:** Hanlin Li, Jiacheng Wang, Yan Zhong, Weiai Liu, Boheng Zheng, Yingzhe Liu, Shicong Niu, Weijun Li, Yu Liu

**Affiliations:** 1 School of Acupuncture, Moxibustion, Tuina and Rehabilitation Medicine, Hunan University of Chinese Medicine, Changsha 410208, China; 202303010624@stu.hnucm.edu.cn (H.L.); 202403010108@stu.hnucm.edu.cn (J.W.); 002115@hnucm.edu.cn (Y.Z.); lwa0433@hnucm.edu.cn (W.L.); 2 School of Mechanical and Automotive Engineering, Xiamen University of Technology, Xiamen 361024, China; 2521011039@stu.xmut.edu.cn (B.Z.); yingzheliu@xmut.edu.cn (Y.L.); 3 Fujian Key Laboratory of Functional Materials and Application, School of Materials Science and Engineering, Xiamen University of Technology, Xiamen 361024, China

**Keywords:** hydrogel, intrauterine adhesion, endometrial regeneration, biomaterials, bioactive delivery

## Abstract

Intrauterine adhesion (IUA) is a fibrotic disorder resulting from aberrant repair following injury to the endometrial basal layer, leading to menstrual abnormalities, infertility, recurrent miscarriage, and pregnancy complications. Although hysteroscopic adhesiolysis remains the primary clinical treatment, the rate of postoperative re–adhesion is still high, especially in patients with moderate–to–severe IUA. Moreover, mechanical separation alone is often insufficient to restore intact endometrial architecture and reproductive function. Hydrogels, with their hydrated three–dimensional networks, extracellular matrix (ECM)–mimicking properties, injectability, biodegradability, tissue adhesion, and tunable delivery capacity, have evolved from passive barrier materials into multifunctional therapeutic platforms capable of regulating the pathological microenvironment and promoting tissue regeneration. Recent advances in responsive, self–healing, adhesive, antioxidant, and bioactive cargo–loaded hydrogels have expanded their applications from preventing adhesion formation toward functional endometrial reconstruction. In this review, we summarize recent progress in hydrogel–based IUA therapy, focusing on material composition, structural design, functional modification, therapeutic mechanisms, and translational considerations. Particular emphasis is placed on disease–informed hydrogel engineering strategies that integrate the unique anatomical characteristics of the uterine cavity, injury–associated microenvironment, and dynamic stages of endometrial repair. This perspective provides insights into the development of next–generation hydrogel systems for preventing re–adhesion and restoring reproductive function.

## 1. Introduction

Intrauterine adhesion (IUA), also known as Asherman syndrome, is a common fibrotic gynecological disease that compromises female reproductive health. It develops following injury to the basal endometrium, where dysregulated repair leads to excessive fibrosis, chronic inflammation, impaired angiogenesis, and defective glandular regeneration, ultimately resulting in partial or complete obliteration of the uterine cavity [1,2]. Intrauterine surgical procedures, chronic infection, and radiotherapy are the main causative factors. Clinically, patients often present with hypomenorrhea, amenorrhea, cyclic lower abdominal pain, recurrent miscarriage, and secondary infertility, which markedly reduce fertility and quality of life [3,4]. Although hysteroscopic transcervical resection of adhesions (TCRA) remains the standard treatment, postoperative re–adhesion occurs in 20–60% of patients, particularly in severe cases [1,5]. Therefore, the major challenge of IUA therapy extends beyond mechanical separation of adhesions, requiring restoration of a vascularized, glandular, and receptive endometrium capable of supporting implantation and pregnancy.

Recent advances in biomaterials and regenerative medicine have provided new opportunities for addressing this challenge. Hydrogels, characterized by hydrated three–dimensional polymer networks, excellent biocompatibility, and tunable physicochemical properties, can partially mimic the natural extracellular matrix (ECM) microenvironment and have emerged as a promising platform for IUA treatment [6,7,8]. Unlike conventional physical barriers, injectable, degradable, adhesive, and responsive hydrogels can be delivered into the uterine cavity through minimally invasive approaches, conform to irregular endometrial wound surfaces, and form a temporary isolation layer to prevent direct contact between injured tissues. Importantly, hydrogel performance is highly dependent on polymer chemistry, crosslinking density, mesh size, swelling behavior, degradation kinetics, tissue adhesion, and release profiles [9,10,11]. These parameters determine whether a hydrogel can maintain uterine retention, match the endometrial regeneration window, avoid excessive compression, and achieve sustained local delivery [12,13]. Therefore, hydrogel design for IUA should be guided by disease–specific requirements rather than simply serving as passive anti–adhesion barriers. Functional hydrogels can also serve as delivery platforms for growth factors, stem cells, exosomes, ABs, platelet–rich plasma (PRP), nucleic acids, or anti–fibrotic drugs, thereby coordinating anti–inflammatory, anti–fibrotic, pro–angiogenic, immunomodulatory, and regenerative effects [14,15,16]. For example, curcumin–loaded carboxymethyl chitosan (CMC)/oxidized hyaluronic acid (HA) hydrogels can downregulate TGF–β1 expression and reverse epithelial–mesenchymal transition (EMT), exerting anti–fibrotic effects. Genetically engineered FGF1–sericin hydrogels inhibit fibrosis by regulating the TGF–β/Smad pathway and promote structural endometrial repair and fertility–related functional recovery in animal models [15].

The rapid evolution of hydrogel–based IUA therapies highlights the need for a systematic understanding of how material design can be matched with disease progression. Emerging hydrogel platforms, including stimuli–responsive, self–healing, antioxidant, adhesive, and bioactive delivery systems, have shifted the field from simple adhesion prevention toward active regulation of the pathological microenvironment. However, effective IUA treatment requires stage–specific intervention. Early after injury, rapid wound coverage, inflammation control, and oxidative stress regulation are critical. During fibrosis progression, inhibition of TGF–β/Smad signaling, myofibroblast activation, and ECMdeposition becomes essential. At later stages, angiogenesis, epithelial restoration, glandular reconstruction, and recovery of endometrial receptivity determine functional outcomes. Thus, establishing a design framework linking “IUA pathological progression–hydrogel properties–therapeutic functions” is essential for developing more precise regenerative strategies.

**Figure 1:**
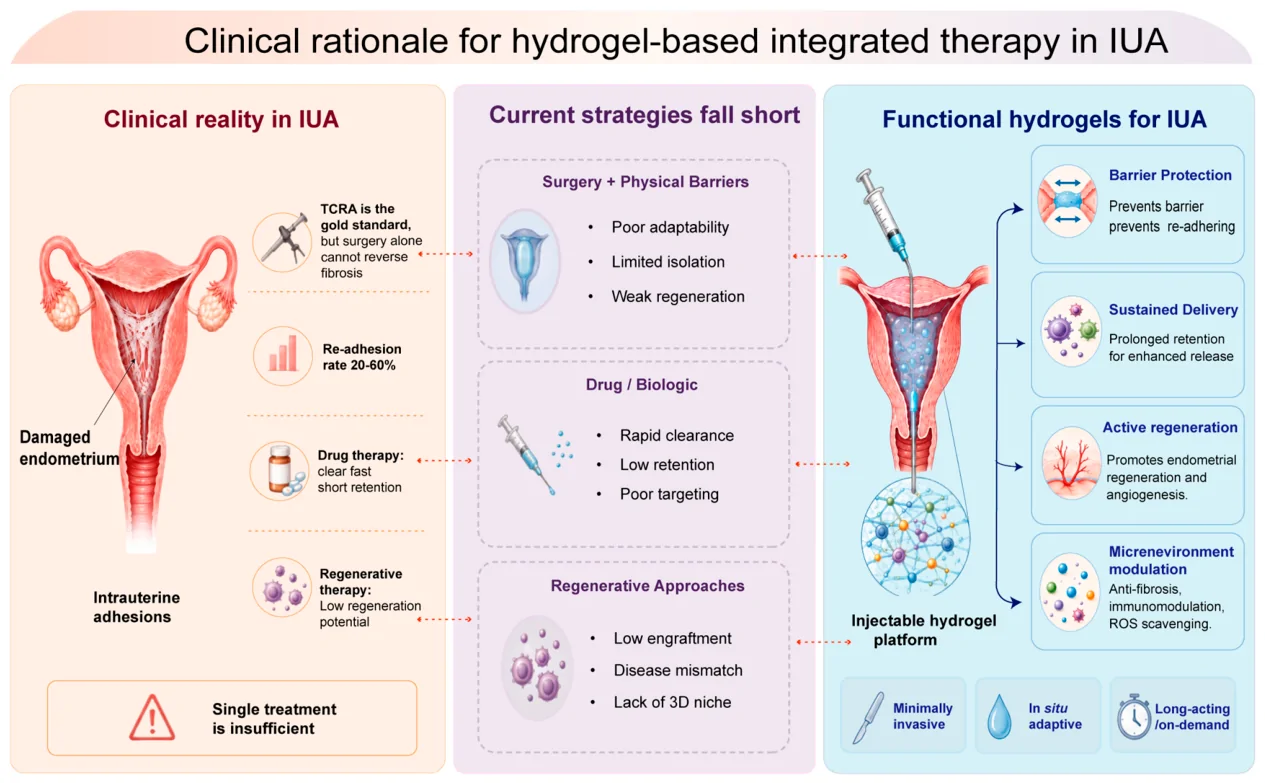
Disease–specific design rationale of multifunctional hydrogel–based platforms for IUA therapy.

Although previous reviews have summarized biomaterial–based strategies for endometrial repair and hydrogel–mediated therapeutic delivery [5,6], they have generally emphasized bioactive cargos and biological outcomes, with less attention to how material properties can be tailored to the pathological and anatomical requirements of IUA. Key demands of the uterine environment—including wet–tissue adhesion, dynamic retention, controlled degradation and release, and reproductive safety—remain insufficiently integrated into hydrogel design. Here, we present a pathology–oriented framework linking specific pathological barriers of IUA with hydrogel physicochemical properties, functional modifications, and therapeutic strategies (Figure 1). We further discuss translational considerations, including gelation behavior, sterilization, manufacturing reproducibility, and fertility–related outcomes, with the aim of guiding the development of hydrogel platforms that achieve both effective re–adhesion prevention and functional endometrial regeneration.

## 2. Pathological Features of IUA and Design Requirements for Hydrogels

### 2.1. Mechanisms of Endometrial Injury and Imbalanced Repair

After normal endometrial injury, orderly repair occurs through epithelial cell proliferation, angiogenesis, and stromal remodeling. In IUA, however, injury to the basal layer of the endometrium disrupts this repair balance, producing a cascade of persistent excessive inflammation, abnormal ECM deposition, insufficient angiogenesis, and destruction of glandular structures, ultimately resulting in fibrotic scar formation and partial or complete uterine cavity occlusion [1,3,5].

Disruption of the post–injury microenvironment is a critical event that triggers imbalanced repair. After intrauterine procedures damage the basal endometrial layer, local oxidative stress increases and reactive oxygen species (ROS) accumulate, aggravating apoptosis and inflammatory cytokine release and creating a pro–fibrotic microenvironment [5,15]. Persistent oxidative and inflammatory stress further activates pro–fibrotic signaling, promotes excessive ECM deposition, and shifts endometrial repair toward scar formation. Recent studies further indicate that endometrial injury is accompanied by abnormal metabolic reprogramming; impaired mitochondrial fatty acid oxidation and carnitine deficiency amplify oxidative stress and fibrotic signaling, establishing a vicious cycle of injury–inflammation–fibrosis [16,17]. In addition, abnormal autophagy and reduced vitamin D receptor (VDR) expression may exacerbate imbalanced repair through different regulatory mechanisms [18,19,20]. These pathological events define the essential design requirements for IUA hydrogels: rapid coverage of injured surfaces, reduction in oxidative and inflammatory stress, sustained inhibition of fibrotic progression, and support for vascularized and glandular endometrial regeneration.

### 2.2. Key Cellular and Molecular Regulatory Networks in Endometrial Fibrosis

Endometrial fibrosis involves coordinated regulation by multiple cell types, factors, and signaling pathways. Fibroblasts are the core effector cells in fibrosis. Under stimulation by TGF–β1, IL–6, and other mediators, they become activated and differentiate into α–SMA–positive myofibroblasts, continuously secreting collagen, fibronectin, and other ECM components, leading to IUA and scar formation [6,21,22]. Imbalanced macrophage polarization is a key immune mechanism driving fibrosis progression. During the early injury phase, M1 macrophages predominate and secrete large amounts of pro–inflammatory factors, including TNF–α, IL–1β, and IL–6, amplifying inflammation and hindering normal repair. Excessive or persistent M2 macrophage activation can also promote fibroblast activation and collagen deposition through TGF–β1 secretion. Together, imbalance between these phenotypes contributes to fibrotic progression [23,24,25].

At the molecular regulatory level, the canonical TGF–β1/Smad pathway is widely recognized as a core fibrotic axis. Activation of downstream Smad2/3 signaling promotes EMT, fibroblast–to–myofibroblast differentiation, and excessive ECM deposition, whereas targeted inhibition of Smad3 can markedly alleviate endometrial fibrosis [2,26]. Non–canonical pathways, including MAPK, PI3K/Akt, and IL–33/ST2/MAPK, further amplify pro–fibrotic signaling through pathway crosstalk [19,20]. Newly identified targets such as THBS1 and MFAP5 accelerate fibrosis by activating TGF–β–related signaling, whereas AMPK/p53/p21 signaling and the FGF family participate in regulatory networks that inhibit fibrosis and promote endometrial repair [16,27,28]. These cellular and molecular mechanisms provide potential targets for hydrogel–mediated anti–fibrotic and immunomodulatory interventions. The pathological cues and corresponding hydrogel design strategies are compiled in Table 1.

### 2.3. Clinical Treatment Bottlenecks and Unmet Clinical Needs

TCRA under hysteroscopy is currently the gold–standard procedure for clinical treatment of IUA, but surgery alone cannot reverse the core pathological process of endometrial fibrosis in the uterine cavity, and clinical outcomes remain unsatisfactory. Data show that postoperative re–adhesion occurs in 20–60% of patients with IUA, and the recurrence rate in severe cases exceeds 60%, highlighting the clear limitations of surgery alone [1,5]. Current postoperative adjuvant interventions all have major shortcomings and cannot fully solve the problems of recurrence and endometrial repair.

In terms of physical barrier interventions, commonly used clinical devices such as intrauterine devices and balloon stents have fixed shapes that poorly match individualized uterine cavity anatomy. They may cause adverse effects such as intrauterine infection and lower abdominal pain, impose compressive injury on the endometrium, and fail to provide a long–term, stable isolation and repair environment within the uterine cavity; therefore, they cannot fundamentally prevent adhesion recurrence [5,29]. Drug therapy also has many limitations. Systemic estrogen therapy, which is commonly used clinically, is associated with significant systemic side effects and insufficient local drug concentration in the uterine cavity, and its repair effect is very limited in patients with severe basal endometrial damage. Traditional anti–fibrotic drugs lack uterine cavity–targeted delivery systems and therefore cannot act precisely on lesions or effectively suppress endometrial fibrosis [29,30].

Emerging regenerative therapies also face clinical bottlenecks that remain difficult to overcome. When stem cells, exosomes, and other regenerative agents are used for IUA treatment, they commonly exhibit low in vivo retention, short survival duration, and lack of a three–dimensional microenvironment suited to uterine cavity repair; when used alone, they cannot achieve stable structural repair and functional reconstruction [31,32,33]. Most importantly, for patients with severe IUA involving extensive adhesions and complete endometrial scarring, no mature and effective standardized treatment is currently available. Existing interventions cannot achieve functional regeneration of the damaged endometrium, and postoperative fertility recovery remains extremely low, making this a major clinical priority and challenge [4].

Commercially available anti–adhesion biomaterials, particularly HA–based gels, have been used as adjunctive barriers after intrauterine surgery and can reduce postoperative adhesion formation [34,35,36]. However, their therapeutic effects remain predominantly preventive rather than regenerative. Their intrauterine retention is formulation–dependent, and current products have limited capacity to actively modulate the oxidative–inflammatory microenvironment, promote angiogenesis, or restore severely damaged basal endometrium [34,35]. Moreover, evidence for improved reproductive outcomes, particularly live birth and miscarriage rates, remains inconclusive [36]. These limitations highlight the need for multifunctional hydrogels that integrate physical isolation with microenvironment regulation and endometrial regeneration.

Clinically, there is an urgent need for an integrated therapeutic platform that combines minimally invasive delivery, in situ adaptation, long–term isolation, targeted drug release, and pro–regenerative functions. Such a platform should match the unique anatomy of the uterine cavity, provide a stable physical isolation barrier, precisely regulate the local uterine cavity microenvironment, effectively inhibit endometrial fibrosis, promote local angiogenesis and functional endometrial regeneration, and address the clinical gap in precise and efficient treatment of severe IUA. Therefore, the evaluation of hydrogel–based therapies should move beyond histological endometrial thickness alone and include adhesion scores, menstrual recovery, uterine cavity morphology, endometrial blood flow, implantation–related markers, clinical pregnancy rate, live birth rate, miscarriage rate, and long–term reproductive safety. Therefore, the therapeutic performance of hydrogel platforms is fundamentally dictated by their intrinsic chemical and micro–structural features, which should be carefully tailored to match the pathological requirements of endometrial injury repair.

### 2.4. Theoretical Rationale and Advantages of Hydrogels as Ideal Platforms for IUA Treatment

Hydrogels can address several major bottlenecks in IUA treatment because their physicochemical and biological properties can be tailored to the specific requirements of uterine cavity repair. Injectable, degradable, and functionalized hydrogels are compatible with the dynamic environment of the uterine cavity and provide a promising platform for combining adhesion prevention with endometrial repair [6,16]. Compared with conventional approaches, their advantages are mainly reflected in physical protection, localized therapeutic delivery, and regulation of the regenerative microenvironment.

For physical protection, injectable hydrogels can be delivered minimally invasively and gel in situ, allowing them to conform closely to irregular endometrial wound surfaces. The resulting hydrogel layer separates opposing injured surfaces and reduces direct wound contact and fibrous bridging during the critical repair period. Their conformability may offer advantages over rigid or pre–shaped intrauterine barriers, although comparative clinical evidence remains limited [5,37]. In addition, hydrogels can serve as local delivery matrices for therapeutic and regenerative agents. Their three–dimensional networks can prolong intrauterine retention and enable sustained release, thereby increasing local bioavailability while reducing systemic exposure [32,38].

Functional modification further expands the role of hydrogels in endometrial repair. Thermosensitive, self–healing, antioxidant, antibacterial, and tissue–adhesive properties can improve their adaptability and retention in the uterine cavity, while bioactive modifications can regulate the pathological microenvironment and support angiogenesis and endometrial reconstruction [39,40]. Preclinical studies have shown that functional hydrogel systems can reduce fibrotic markers, improve endometrial regeneration, and enhance fertility–related outcomes [6,32].

Overall, hydrogels provide a versatile platform for integrating physical isolation, localized therapeutic delivery, and regenerative intervention in IUA treatment. However, these therapeutic advantages depend strongly on material properties such as tissue adhesion, mechanical characteristics, swelling behavior, degradation kinetics, and release profiles. Rational optimization of these parameters is therefore essential to ensure that hydrogel performance is appropriately matched to the requirements of intrauterine application.

## 3. Material Categories and Modification Strategies of Hydrogels for IUA Treatment

Based on material source and chemical structure, hydrogels currently used for IUA treatment can be divided into four categories: natural polymer hydrogels, synthetic polymer hydrogels, composite hydrogels, and intelligent responsive hydrogels [41,42,43]. These systems differ markedly in injectability, tissue adhesion, degradation rate, drug–loading capacity, and pro–regenerative activity. Rational material selection and structural design are essential for achieving long–term physical isolation, precise drug delivery, and efficient endometrial regeneration [44,45]. This section systematically describes the composition, properties, advantages, and improvement strategies of different hydrogel systems by material category and further outlines the design logic of intelligent responsive hydrogels, laying a materials–science foundation for the functional applications and mechanistic discussion that follow [46,47]. A summary of polymeric materials used in hydrogel systems is provided in Table 2.

### 3.1. Natural Polymer Hydrogels

**Figure 2:**
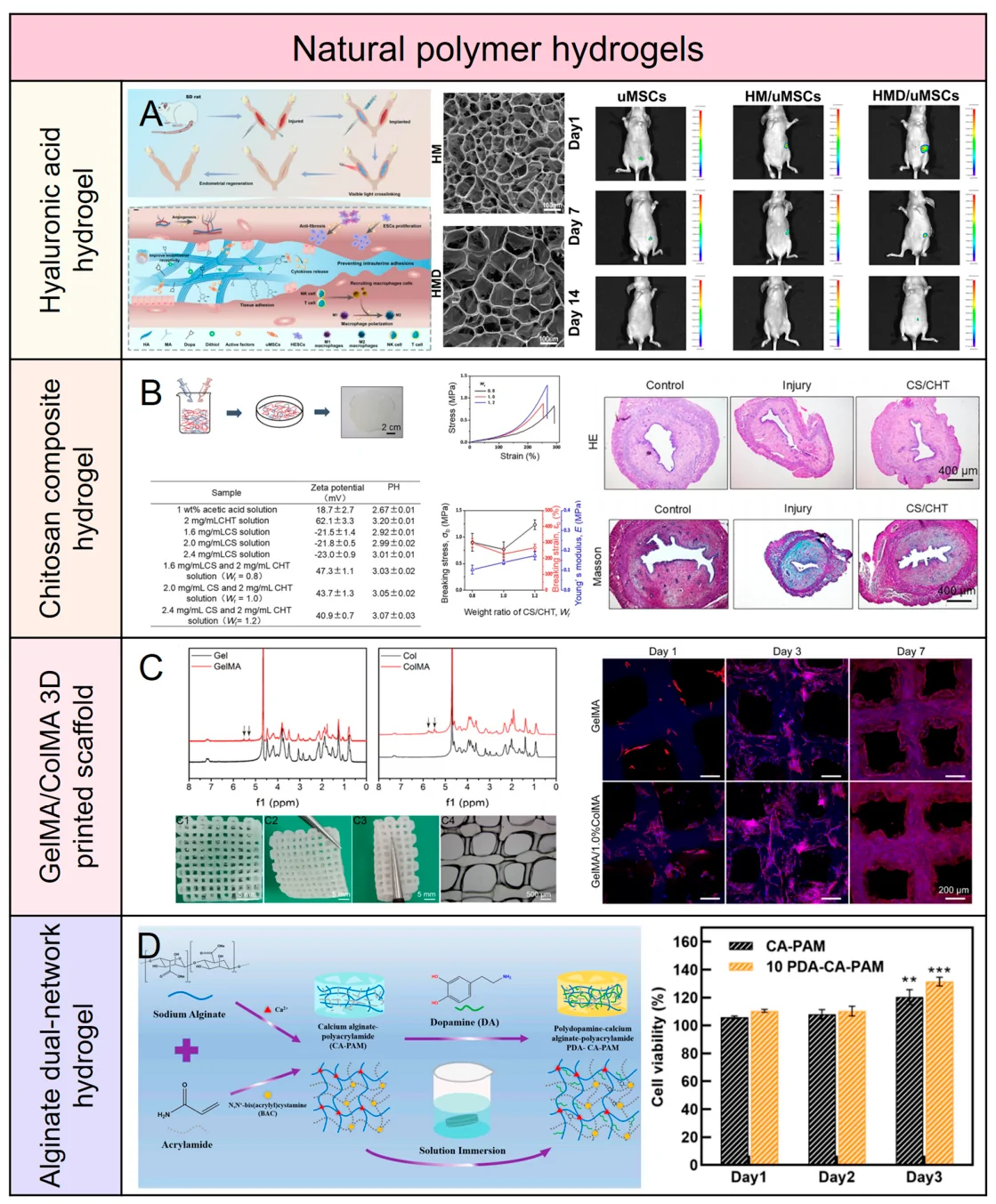
Fabrication, characterization, and biological evaluation of representative natural polymer hydrogels for IUA therapy. (**A**) Porous structure and 14–day in vivo retention of a stem cell–laden HA hydrogel. Reproduced with permission [48]. (**B**) Fabrication, mechanical characterization, and uterine histological evaluation of a chitosan–based composite hydrogel. Reproduced with permission [53]. (**C**) Structural characterization, 3D scaffold morphology, and cell adhesion of a GelMA/ColMA hydrogel. Reproduced with permission [60]. (**D**) Synthesis and in vitro cell viability evaluation of a dopamine–modified alginate dual–network hydrogel. ** indicates *p* < 0.01, and *** indicates *p* < 0.001. Reproduced with permission [68].

Natural polymer hydrogels are derived from animal, plant, or microbial sources and mainly include polysaccharide– and protein–based materials. Representative systems used for IUA therapy include HA, chitosan, gelatin, collagen, alginate, fibrin, silk fibroin, and decellularized ECM–derived hydrogels. Owing to their structural similarity to native ECM, abundant hydrophilic groups, favorable cytocompatibility, and biodegradable characteristics, natural polymer hydrogels have become one of the most widely investigated material classes for postoperative adhesion prevention, structural endometrial repair, and functional endometrial regeneration. Their biological advantages include supporting endometrial epithelial, stromal, and endothelial cell adhesion, proliferation, and migration, reducing foreign–body responses, and providing a permissive microenvironment for immune regulation and vascular remodeling. However, natural polymers also have inherent limitations, including weak mechanical strength, rapid enzymatic degradation, insufficient uterine retention, batch–to–batch variability, and difficulty in precisely controlling swelling, degradation, and release kinetics. Therefore, their use in IUA therapy usually requires rational material engineering, including chemical modification, dynamic covalent crosslinking, ionic coordination, photo–crosslinking, thermosensitive composite formation, double–network construction, nanoparticle incorporation, and bioactive cargo loading. These strategies aim to balance biocompatibility, wet adhesion, mechanical adaptability, degradation window, and reproductive safety within the dynamic uterine cavity. Representative natural polymer hydrogels are illustrated in Figure 2. Cross–linking density of these natural polymer networks directly modulates pore dimension, mechanical stiffness and degradation profile, which further regulate mass transport and cell–matrix interaction. 

#### 3.1.1. Hyaluronic Acid–Based Hydrogels

HA is an important component of the endometrial ECM and can regulate cell behavior, inflammatory responses, tissue hydration, and matrix remodeling by interacting with cell–surface receptors such as CD44 and RHAMM. Therefore, HA–based hydrogels combine physical barrier function with biological signaling regulation and have been widely used in intrauterine anti–adhesion and endometrial regeneration studies [48]. In the uterine cavity, HA hydrogels can rapidly cover irregular wound surfaces, separate injured tissues, reduce fibrin bridging, and provide a temporary matrix–like environment for epithelial coverage and stromal remodeling [49]. In addition to simple crosslinked HA gels, several material design strategies have been developed to improve their performance. Methacrylated HA enables light–mediated crosslinking and tunable network formation; oxidized HA can react with amino–containing polymers such as CMC or gelatin through Schiff–base bonds to form injectable and self–healing dynamic networks; dopamine–grafted HA enhances wet tissue adhesion through catechol–mediated interfacial interactions; and HA–based composite systems can be combined with nanoparticles, stem cells, exosomes, ABs, or anti–fibrotic drugs to achieve multifunctional therapy [39]. For IUA therapy, HA–based hydrogels are particularly suitable for early postoperative wound coverage and local delivery because of their excellent mucosal compatibility and established anti–adhesion background. HA hydrogels loaded with mesenchymal stem cells (MSCs), exosomes, or ABs can improve the local regenerative microenvironment, promote macrophage polarization toward a reparative phenotype, suppress fibrosis, and enhance angiogenesis [50,51,52]. Nevertheless, several IUA–specific problems remain. Unmodified HA hydrogels usually have weak mechanical strength, rapid degradation, limited wet adhesion, and poor resistance to uterine fluid dilution and uterine peristalsis, which may lead to premature loss before completion of the high–risk re–adhesion window. Excessive swelling may also increase intrauterine pressure and affect tissue recovery. In addition, photo–crosslinked HA systems require careful evaluation of photoinitiator toxicity and light exposure safety in reproductive tissues. Therefore, HA–based systems should be evaluated not only for cytocompatibility but also for gelation time, wet adhesion to injured endometrial surfaces, uterine retention, degradation within the re–epithelialization window, controlled release behavior, and compatibility with embryo implantation after material clearance.

#### 3.1.2. Chitosan–Based Hydrogels

Chitosan is a cationic polysaccharide derived from deacetylated chitin. Its positively charged chains can interact electrostatically with negatively charged mucosal tissues and ECM components, thereby facilitating local retention. Chitosan also possesses antibacterial, hemostatic, and anti–inflammatory properties, making it attractive for the early inflammatory phase after intrauterine surgery [53]. In IUA therapy, chitosan–based hydrogels may therefore provide both local retention and a favorable microenvironment for endometrial repair [54]. Chemical modification and crosslinking strategies have been developed to overcome the limited physiological solubility and mechanical stability of native chitosan. Hydrophilic chitosan derivatives improve aqueous solubility, while dynamic covalent crosslinking, particularly through reversible imine bonds, enables injectable and self–healing network formation [55]. Chitosan/β–glycerophosphate (CS/β–GP) systems provide an alternative thermoresponsive strategy, undergoing sol–gel transition near physiological temperature for in situ hydrogel formation [56].

PRP–containing hydrogel systems also illustrate the potential of bioactive hydrogel matrices for IUA treatment. Platelet–rich plasma–loaded injectable double–network hydrogels can provide a localized regenerative microenvironment, enhance endometrial regeneration, and improve reproductive outcomes after intrauterine injury [57].Thermosensitive chitosan/sodium alginate/β–glycerophosphate hydrogels can rapidly gel at body temperature and, after aspirin loading, inhibit TGF–β1/Smad signaling, reduce fibrotic marker expression, and promote angiogenesis [37]. Moreover, the antibacterial and hemostatic properties of chitosan may be particularly beneficial during the early postoperative inflammatory phase [58].

Despite these advantages, chitosan–based hydrogels have several IUA–specific limitations. Native chitosan has poor solubility at neutral pH, while its cationic nature requires careful optimization of the degree of deacetylation, molecular weight, and charge density to avoid excessive tissue interactions. Some chitosan hydrogels also exhibit insufficient mechanical stability and rapid erosion under wet conditions. Strong electrostatic adhesion may improve intrauterine retention but could potentially interfere with epithelial repair or material clearance. Therefore, future chitosan–based hydrogels should balance antibacterial activity, wet adhesion, mechanical stability, degradation, and reproductive safety, particularly when used for the delivery of PRP, drugs, or extracellular vesicles (EVs).

#### 3.1.3. Gelatin– and Collagen–Based Hydrogels

Collagen is the major structural protein of the ECM, while gelatin is its partially hydrolyzed derivative with improved solubility and processability. Both materials contain abundant cell–interactive motifs, including RGD sequences, which support endometrial cell adhesion, spreading, proliferation, and matrix remodeling [59].Therefore, collagen– and gelatin–based hydrogels are highly suitable for constructing ECM–mimetic scaffolds and cell–delivery platforms for IUA therapy. Their material design strategies mainly include physical thermal gelation, chemical crosslinking, methacrylation, enzymatic crosslinking, dynamic covalent crosslinking, 3D bioprinting, and composite network formation. Gelatin methacryloyl (GelMA), in particular, enables photocontrolled crosslinking and precise regulation of pore size, stiffness, degradation rate, and cell encapsulation capacity [60].

In IUA–related studies, gelatin– and collagen–based hydrogels have mainly been used as tissue–engineering scaffolds and stem–cell delivery matrices. A 3D–printed scaffold composed of GelMA and poloxamer can efficiently load adipose–derived MSCs; its stable porous structure improves cell retention and promotes angiogenesis, glandular reconstruction, and fibrosis alleviation [61]. Gelatin–based colloid–assembled self–healing (CASH) hydrogels exhibit shear–thinning and minimally invasive implantation properties, mimic the ECM microenvironment, and enhance stem–cell paracrine function, thereby supporting functional endometrial regeneration through coordinated immune remodeling, fibrosis resolution, and vascular regeneration [62]. Because of their high biomimicry and strong cell affinity, collagen– and gelatin–based hydrogels are promising scaffolds for stem–cell delivery, endometrial tissue engineering, and reconstruction of glandular and vascular structures [63]. However, their application in IUA still faces important limitations. Unmodified gelatin and collagen hydrogels often show weak mechanical strength, rapid enzymatic degradation, thermal instability, and limited retention in the uterine cavity. GelMA systems require photoinitiators and light exposure, raising concerns regarding cytotoxicity, incomplete polymerization, and reproductive tissue safety. In addition, collagen–based materials may have source–dependent immunogenicity, pathogen transmission risk, and batch variability. Therefore, collagen– and gelatin–based hydrogels should be optimized for mild crosslinking, controlled degradation, cell viability after encapsulation, wet retention, and long–term reproductive safety before clinical translation.

#### 3.1.4. Alginate–Based Hydrogels

Alginate is a naturally derived anionic polysaccharide mainly composed of β–D–mannuronic acid and α–L–guluronic acid units. It can be mildly crosslinked by divalent cations such as Ca^2+^ through the classical “egg–box” model, offering simple gelation conditions, good biocompatibility, and high drug–loading capacity [64]. Because of its mild ionic gelation and favorable encapsulation properties, alginate can serve as an injectable matrix for local delivery of regenerative cells such as UCMSCs [65]. For IUA therapy, alginate–based hydrogels can be designed as injectable bulk gels, thermosensitive composite gels, oxidized alginate dynamic networks, strontium– or calcium–coordinated hydrogels, microfluidic porous scaffolds, microcapsules, or double–network systems. Oxidized sodium alginate (SA) can form Schiff–base bonds with amino–containing polymers such as chitosan or gelatin, enabling injectable, self–healing, and degradable networks.

Representative alginate–based systems have shown therapeutic potential in IUA models. Multifunctional hydrogels constructed from oxidized SA, strontium, and betamethasone can promote endothelial cell migration, inhibit fibrosis, increase endometrial thickness and gland number, and improve fertility–related outcomes [66]. Thermosensitive SA/chitosan composite hydrogels can rapidly gel in situ and form stable porous structures, combining physical isolation, anti–fibrotic activity, and regenerative support through co–delivered anti–inflammatory drugs [37]. Alginate–based hydrogels also have potential translational advantages because of their low cost, scalability, mild gelation, and strong drug–loading capacity [67]. However, several limitations must be considered in IUA therapy. Alginate lacks intrinsic cell–adhesion motifs, which may restrict endometrial epithelial and stromal cell attachment unless modified with RGD peptides, gelatin, collagen, or ECM–like components. Ionically crosslinked alginate gels may undergo ion exchange in physiological fluids, leading to uncontrolled swelling, premature weakening, or unstable degradation. Excessive swelling could create pressure within the small uterine cavity, while slow degradation or residual gel may interfere with embryo implantation. Furthermore, the mechanical properties and degradation rate of alginate hydrogels strongly depend on alginate composition, molecular weight, guluronic acid content, ion type, and crosslinking density. Therefore, future alginate–based hydrogels for IUA should focus on improving cell–interactive bioactivity, controlling swelling behavior, matching degradation with the postoperative repair window, and ensuring complete clearance before the implantation period.

### 3.2. Synthetic Polymer Hydrogels

**Figure 3:**
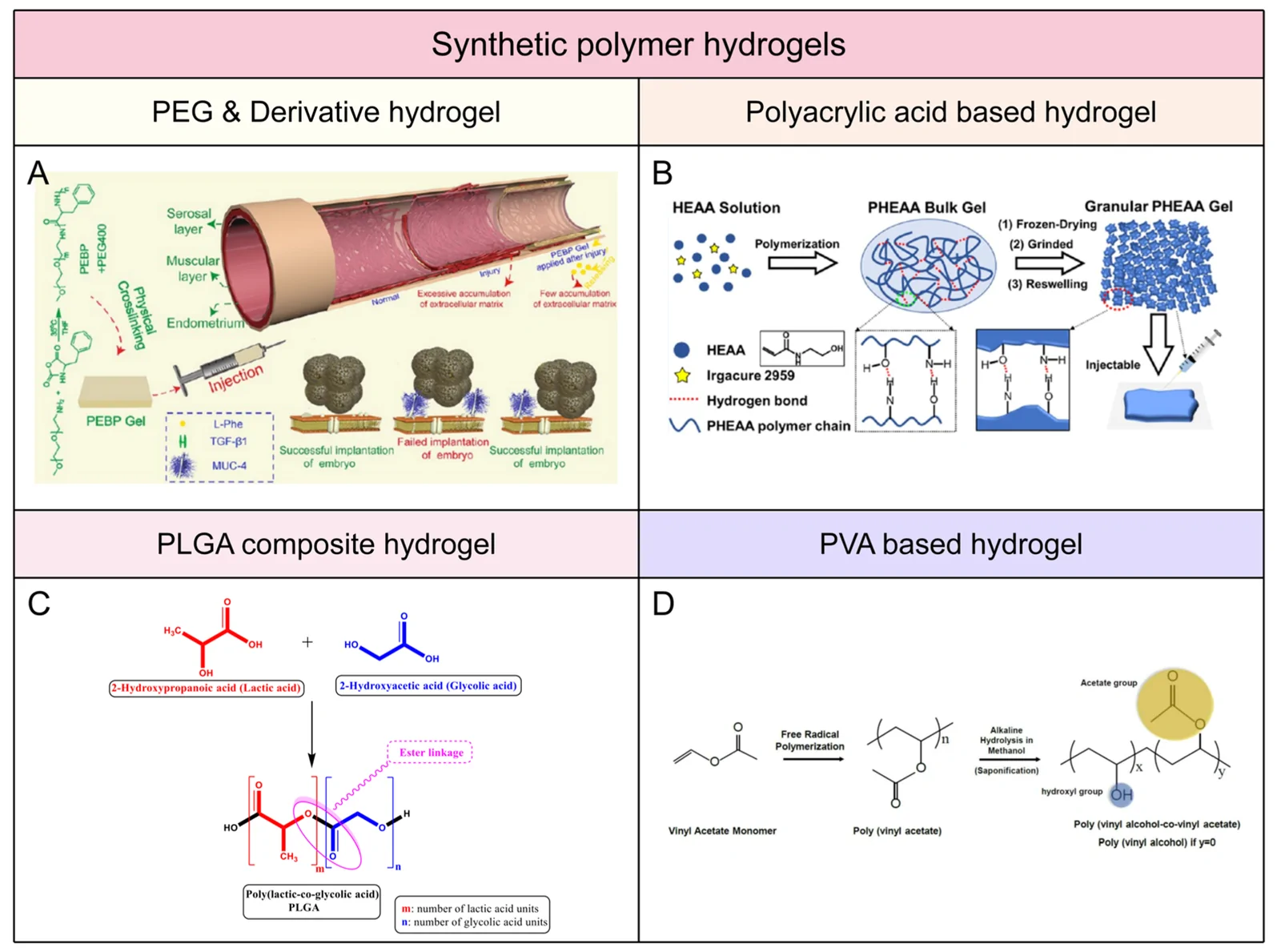
Design, fabrication, and functional characteristics of representative synthetic polymer hydrogels for IUA therapy. (**A**) Crosslink–tunable peptide–grafted PEG hydrogel. Reproduced with permission [26]. (**B**) Tough adhesive polyacrylic acid–based double–network hydrogel. Reproduced with permission [77]. (**C**) Degradable PLGA–based composite hydrogel for sustained cargo delivery. Reproduced with permission [78]. (**D**) Physically crosslinked PVA hydrogel fabricated by freeze–thaw cycling. Reproduced with permission [79].

Synthetic polymer hydrogels offer prominent advantages, including precisely controllable structure, stable mechanical properties, good batch consistency, and adjustable degradation rate. They can compensate for the weak mechanics, enzymatic susceptibility, and poor stability of natural materials, and are core backbones for constructing long–acting physical barriers and controlled delivery systems. Compared with natural polymers, synthetic materials are more bioinert and less immunogenic, but lack cell–recognition sites and biological signals. Functional modification is therefore required to confer targeted adhesion, drug–loading, and pro–regenerative abilities. As a result, the design paradigm of synthetic backbone + natural functionalization has become mainstream for high–performance uterine cavity repair hydrogels. Figure 3 shows the design and characteristics of typical synthetic polymer hydrogels.

#### 3.2.1. Polyethylene Glycol (PEG) and Derivative Hydrogels

PEG is one of the most widely used synthetic hydrophilic polymers in biomedicine and is characterized by low protein adsorption, low immunogenicity, high hydrophilicity, and programmable structure [2]. The mechanical strength, swelling ratio, and degradation period of PEG hydrogels can be precisely regulated by molecular weight, crosslinking density, and functional–group structure, making them ideal physical anti–adhesion barriers [26]. PEG derivatives such as poly(2–hydroxyethyl methacrylate) (PHEMA) can achieve greatly improved stability through double–network design, remaining stable in uterine fluid for more than 30 days. Their mechanical properties can be comparable to those of intrauterine devices, and after estrogen loading they can provide sustained release and promote endometrial regeneration [30]. PEG can also be end–modified to graft RGD peptides, antibodies, or growth factors, enabling targeted adhesion and precise regulation while reducing foreign–body reactions [69]. With high stability, programmability, and low immunogenicity, PEG–based hydrogels have become a core platform for advanced intelligent anti–adhesion materials [70].

#### 3.2.2. Polyacrylic Acid and Derivative Hydrogels

Polyacrylic acid hydrogels have high water content, high adhesiveness, and excellent mechanical tunability. Copolymerization with other monomers can generate high–strength and high–toughness double–network systems [68]. Composite formation between polyacrylic acid and alginate, chitosan, or other polymers can markedly improve structural stability and tissue adhesion while preserving good biocompatibility [71]. Some polyacrylic acid derivatives can load and release drugs through electrostatic interactions and are suitable as delivery carriers for anti–inflammatory and anti–fibrotic small molecules [72]. These materials show high toughness, strong deformation adaptability, and good biosafety as postoperative anti–adhesion barriers, making them suitable for the dynamic mechanical environment of the uterine cavity [73].

#### 3.2.3. Polyvinyl Alcohol (PVA)–Based Hydrogels

PVA has high hydrophilicity, high mechanical strength, and low immunogenicity. It can form physically crosslinked hydrogels through repeated freeze–thaw cycles without toxic chemical crosslinkers [74]. PVA hydrogels exhibit high mechanical strength, friction resistance, and stability, and can be used as high–strength physical barriers for patients at high risk of recurrence [75]. Composite formation with natural polymers can improve insufficient cell affinity, enabling synergy between “high strength” and “high bioactivity” [76]. PVA–based hydrogels have unique application potential in uterine cavity repair scenarios that require strong mechanical support and long–term isolation [40]. Therefore, synthetic hydrogels are more suitable as mechanically stable and programmable frameworks, whereas their use for IUA generally requires biofunctionalization with adhesive motifs, degradable linkers, ECM–mimetic components, or bioactive cargos.

### 3.3. Intelligent Responsive Hydrogels

**Figure 4:**
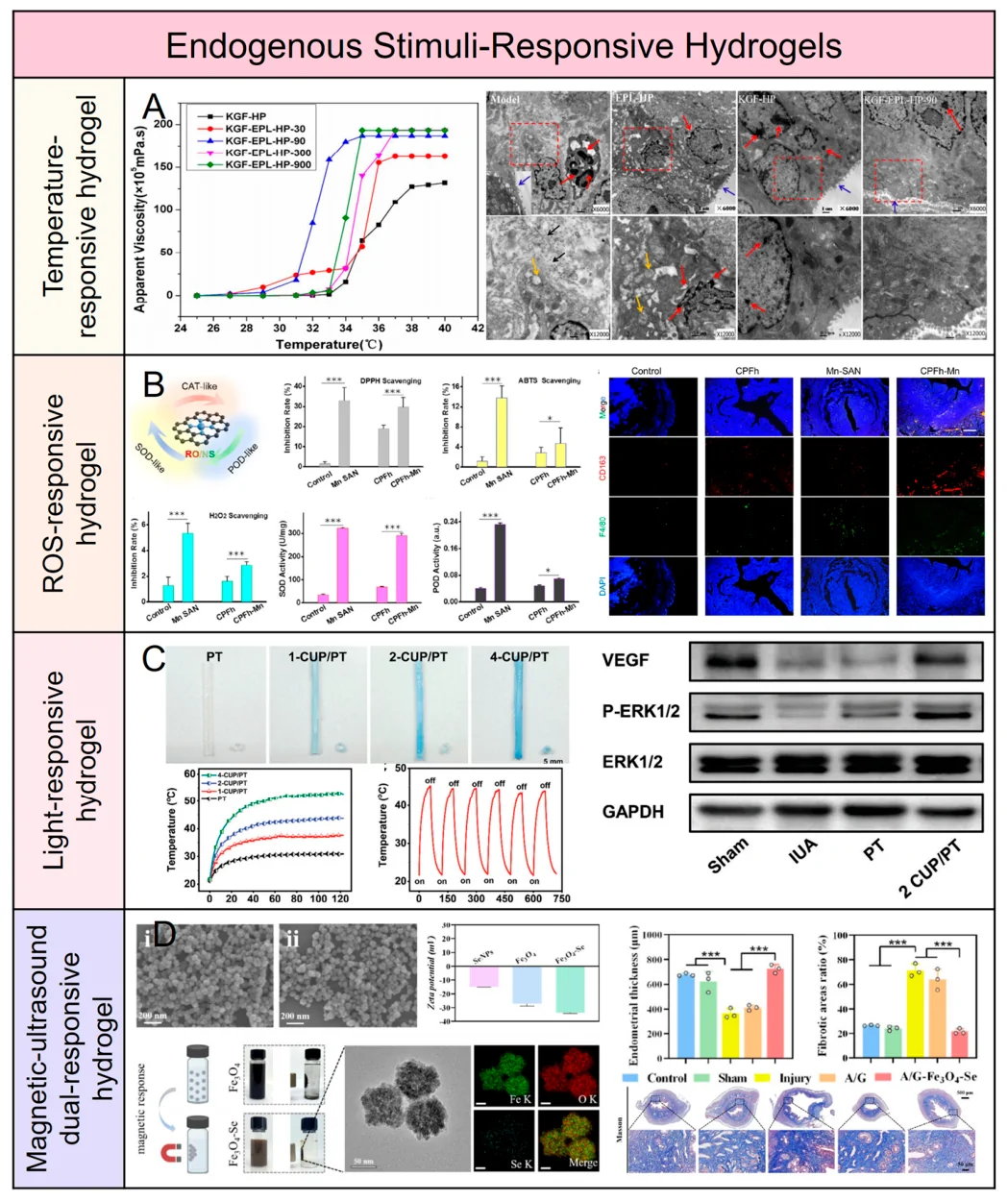
Responsive properties and biological evaluation of representative stimuli–responsive hydrogel systems for IUA therapy. (**A**) Thermoresponsive rheological behavior and uterine histological evaluation of a KGF–loaded composite hydrogel. Reproduced with permission [87]. (**B**) ROS–scavenging activity and uterine immunofluorescence evaluation of a Mn single–atom hydrogel. * indicates *p* < 0.05, and *** indicates *p* < 0.001. Reproduced with permission [94]. (**C**) Shape–memory behavior, photothermal properties, and repair–related protein expression of an NIR–responsive scaffold. Reproduced with permission [91]. (**D**) Physicochemical characterization, magnetic responsiveness, and in vivo therapeutic evaluation of magnetic–ultrasound–responsive microcapsules. *** indicates *p* < 0.001. Reproduced with permission [93].

Intelligent responsive hydrogels can sense multiple microenvironmental signals in the uterine cavity, including pH, temperature, redox state, ROS, and magnetic fields, to precisely modulate gelation, drug release, structural deformation and degradation. These properties significantly enhance the targeting and therapeutic efficacy of uterine cavity treatment [80]. Based on signal sources, these hydrogels can be classified into two categories: endogenous physiological/pathological stimuli–responsive hydrogels that respond to in vivo microenvironmental changes, and exogenous physical stimuli–responsive hydrogels that respond to external man–made physical triggers. The responsive behaviors of representative intelligent hydrogel systems are summarized in Figure 4.

#### 3.3.1. Endogenous Stimuli–Responsive Hydrogels

Endogenous stimuli–responsive hydrogels exploit physiological or pathological signals within the injured uterine microenvironment, primarily temperature, ROS, and local pH fluctuations, to regulate gelation, degradation, or therapeutic release.

Uterine cavity injury induces massive ROS accumulation and oxidative stress, which serves as a core pathological microenvironmental feature of IUA. ROS–responsive hydrogels contain functional moieties such as disulfide bonds and phenylboronic esters, which enable responsive degradation and targeted drug release at injured sites [81,82]. This behavior effectively scavenges excessive ROS and remodels the impaired oxidative microenvironment [83]. Such hydrogels can precisely target damaged tissues, alleviate inflammation, inhibit ferroptosis, and facilitate endometrial epithelial regeneration [84]. Moreover, single–atom nanozyme composite hydrogels can mimic antioxidant enzyme activities, efficiently eliminate ROS, and block fibrotic signaling pathways, providing promising effects for IUA treatment [18].

Temperature–responsive hydrogels represent a typical endogenous physiological responsive material for uterine cavity therapy. The sol–gel transition of poloxamer PF–127 relies on the physiological body temperature of 37 °C in vivo, rather than external physical stimulation. These hydrogels remain fluid at low temperatures for convenient injection and undergo rapid in–situ gelation triggered by intrauterine physiological temperature, enabling non–invasive injection, in–situ cavity filling and adaptive fitting to irregular uterine structures [85]. This property greatly improves clinical operability and patient comfort. However, pure thermosensitive hydrogels possess weak mechanical properties, which can be effectively strengthened by composite incorporation of GelMA, collagen or chitosan [86]. In addition, heparin–poloxamer thermosensitive systems can tightly bind growth factors, prolong intrauterine retention time, and improve endometrial repair outcomes [87].

Dynamic pH fluctuations occur at uterine injury sites after surgery. Unlike tumors or severe infected wounds characterized by dramatic acidification, the intrauterine cavity tends to maintain relatively stable pH owing to the buffering capacity of exudate. Consequently, only focal mild acidification arises within local inflammatory lesions, which may act as an auxiliary trigger for pH–responsive hydrogels. Hydrogels modified with protonatable groups exhibit increased swelling and accelerated drug release under an acidic microenvironment, thereby exerting therapeutic effects specifically at injury sites [88,89]. Such materials are suitable for delivering anti–inflammatory and anti–fibrotic agents to inflamed regions, elevating local drug concentration while minimizing systemic exposure [90].

#### 3.3.2. Exogenous Stimuli–Responsive Hydrogels

Exogenous stimuli–responsive hydrogels use externally applied physical signals, such as magnetic fields and near–infrared (NIR) light, to regulate material localization, structural transformation, or therapeutic release.

Magnetic–responsive hydrogels enable external magnetic–guided auxiliary regulation for intrauterine therapy. Different from uniform distribution, applied magnetic fields can guide magnetic hydrogel components to achieve targeted anchoring at specific lesion sites and local accumulation, avoiding material loss in irregular uterine cavities. This magnetic targeting effect effectively improves the retention and local repair efficiency of therapeutic materials at injured endometrial regions [25].

NIR light–responsive hydrogels allow external optical control for precise therapy. NIR–II photothermal composite hydrogels can trigger shape recovery and controlled drug release upon exogenous light irradiation. This responsive behavior improves the hydrogel’s adhesion and filling performance on irregular wound surfaces, realizing accurate targeted delivery and optimized uterine repair [91,92].

Recently, multi–responsive microcapsules have further advanced the application of intelligent hydrogels in IUA treatment. A/G–Fe_3_O_4_–Se microcapsules consist of an alginate/GelMA double–network hydrogel shell, magnetic Fe3O4–Se nanoparticles, and an ultrasound–responsive liquid core, which can be uniformly fabricated via microfluidic technology [93]. This integrated system combines exogenous magnetic and ultrasound–guided regulation with endogenous temperature–triggered gelation and antioxidant pro–repair functions. It adapts well to irregular intrauterine microenvironments, reduces oxidative stress, promotes endometrial regeneration, improves endometrial receptivity, and enhances pregnancy outcomes in IUA rat models. Collectively, intelligent hydrogels have evolved from single–modal response to combined endogenous–exogenous responsive systems, integrating in vivo physiological/pathological self–response and external precise controllability to achieve superior uterine cavity repair performance.

Together with adhesion, mechanical properties, porosity, and degradation behavior discussed above, stimulus responsiveness constitutes an important material–level determinant of hydrogel performance in IUA therapy. How these physicochemical features translate into specific therapeutic functions will be discussed in Section 4.

## 4. Intrinsic Functionalities of Hydrogel Platforms for IUA Therapy

Section 3 has catalogued various hydrogel matrices and their physicochemical properties, yet Section 2.3 notes that conventional hydrogels cannot reverse IUA pathology. This section focuses on how intrinsic hydrogel functionalities translate into therapeutic effects in IUA, linking injectability, self–healing, tissue adhesion, stimulus responsiveness, and bioactivity with anti–adhesion, microenvironment regulation, and endometrial regeneration. Key parameters such as tissue adhesion, mechanical strength, pore architecture, and degradation kinetics collectively influence fibrosis, inflammation, angiogenesis, and epithelial regeneration, ultimately determining reproductive recovery.

### 4.1. Injectable and Self–Healing Hydrogels

Injectability and self–healing enable hydrogels to conform to irregular intrauterine wounds after minimally invasive administration and maintain barrier integrity under uterine contractions. These properties improve intrauterine retention and provide continuous separation of injured surfaces during the critical postoperative repair period.

These materials possess inherent radical–scavenging ability and can directly neutralize excessive ROS produced after uterine cavity injury, which has been reported to reduce oxidative–stress–related markers in preclinical IUA models. In animal models of IUA, these hydrogels regulate fibrosis through their own structure, downregulate abnormal expression of TGF–β1 and vascular endothelial growth factor, inhibit excessive proliferation of fibrotic scar tissue, and prevent IUA through both physical isolation and microenvironmental regulation [33]. Another study constructed a natural polysaccharide–based injectable self–healing hydrogel through dynamic Schiff–base crosslinking. Even without exogenous active substances, it effectively promotes repair and angiogenesis in injured endometrial tissue [75]. In addition, injectable dynamic hydrogels constructed from oxidized dextran and lysozyme also possess intrinsic injectability and self–healing properties. They can be delivered into the uterine cavity using a minimally invasive syringe and rapidly self–crosslink to form a protective barrier [95]. These studies demonstrate that injectable and self–healing functional hydrogels can rely on intrinsic structural advantages to adapt to uterine physiological features and exert basic therapeutic effects without additional functional loading.

Beyond simple injectability and self–healing capacity, suitable porosity can facilitate cell infiltration and epithelial regeneration, while appropriate mechanical strength helps maintain barrier integrity without excessive compression of the residual endometrium. Inadequate mechanical stability may lead to premature material breakdown, interrupting anti–adhesive protection and potentially impairing subsequent angiogenesis and tissue repair. Therefore, the core design goal is to balance injectability with stable intrauterine retention through dynamic reversible interactions.

### 4.2. Tissue–Adhesive Hydrogels

Tissue adhesion improves intrauterine retention by anchoring hydrogels to wet endometrial surfaces despite uterine fluid and mechanical disturbance. Molecular modification that enhances intrinsic tissue adhesion can therefore prolong local residence and maintain effective hydrogel coverage during postoperative endometrial repair.

The snail–mucus–inspired GS hydrogel relies on multimodal interfacial interactions to firmly anchor onto endometrial wounds and resist flushing–induced loss [39]. Dopamine–modified HA–based GHD hydrogel (see Section 3.1.1) likewise achieves robust wound–surface fixation after minimally invasive delivery [87]. The catechol–containing Janus OD/GM@PG hydrogel further improves interfacial toughness through capillary–assisted wet adhesion [73]. These adhesive systems demonstrate that enhanced interfacial interactions can improve hydrogel stability on injured endometrial surfaces without relying on additional cargo loading.

Robust wet–tissue adhesion is more than a material property; by prolonging hydrogel residence within the dynamically contracting uterine cavity, it helps maintain continuous separation of injured surfaces and reduce fibrous bridging during postoperative repair. Stable local retention may also provide a favorable environment for epithelial reconstruction, thereby supporting subsequent functional endometrial recovery.

### 4.3. Temperature–Responsive Intelligent Hydrogels

As described in Section 3.3.1, thermoresponsive hydrogels undergo a body–temperature–driven sol–gel transition. For IUA therapy, this property facilitates minimally invasive administration, conformal coverage of injured endometrial surfaces, and prolonged intrauterine residence after gelation. Beyond convenient in situ gelation, their degradation rate and post–gelation stability need to be carefully tuned to synchronize with endometrial repair, thereby maintaining effective physical protection during the critical wound–healing period.

A temperature–responsive hydrogel constructed from a Pluronic HP407/HP188 polymer matrix and recombinant type III collagen (rCol III) rapidly forms a conformal hydrogel after intrauterine administration, providing local coverage and structural support for injured endometrial tissue [73]. Heparin–poloxamer thermosensitive hydrogels similarly maintain structural stability after in situ gelation and adapt to the dynamic morphology of the uterine cavity. Their intrinsic anti–inflammatory and antioxidant activities can further regulate the local microenvironment, reduce excessive inflammatory responses, and create favorable conditions for physiological endometrial repair [88]. In addition, chitosan/β–glycerophosphate (CS/GP) thermosensitive hydrogels form flexible networks that adapt to uterine contractions and improve local retention, providing a potential platform for subsequent delivery of therapeutic factors [96]. Silk fibroin/poloxamer (SF/PF127) thermosensitive hydrogels combine conformal wound coverage with the biocompatibility of silk fibroin, providing physical support and a suitable microenvironment for functional endometrial repair [97].

**Figure 5:**
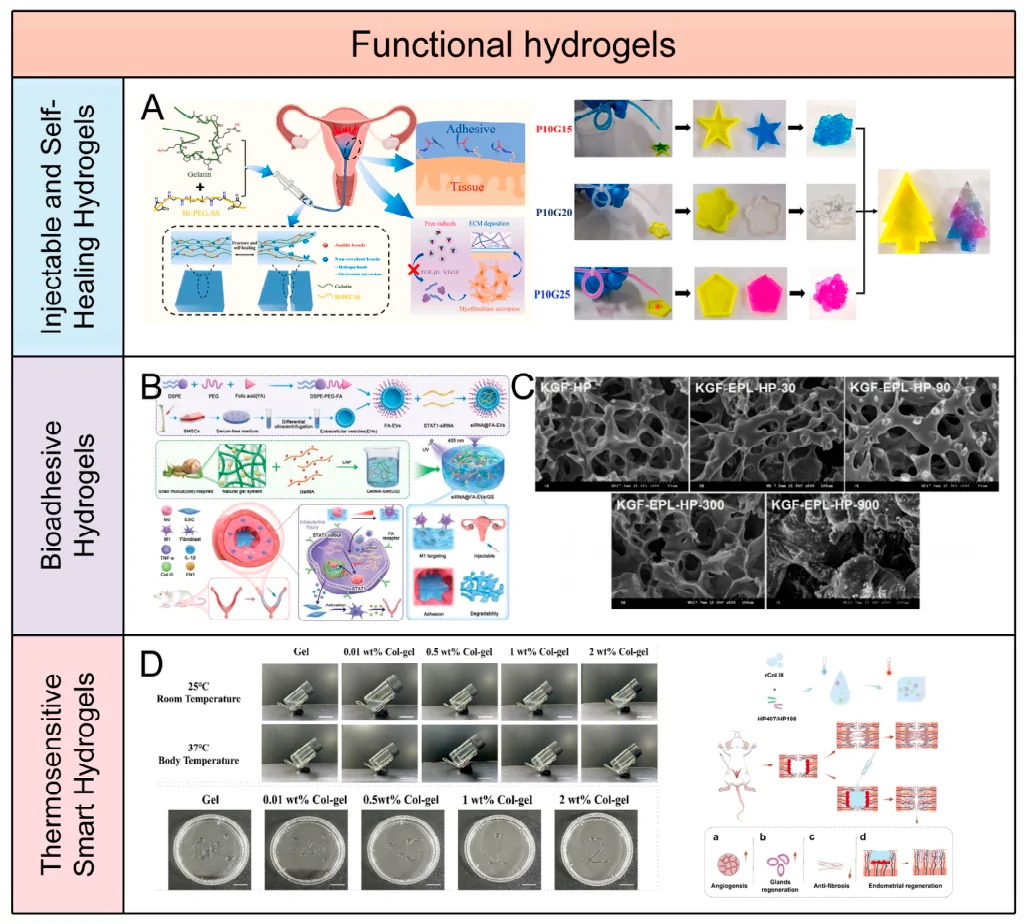
Functional properties and therapeutic applications of representative hydrogel platforms for IUA therapy. (**A**) Design, intrauterine administration, and characterization of an injectable self–healing hydrogel. Reproduced with permission [33]. (**B**) Fabrication, tissue–adhesion mechanism, and endometrial repair evaluation of a snail–mucus–inspired hydrogel. Reproduced with permission [39]. (**C**) SEM characterization of the porous network structures of bioadhesive hydrogels with different formulations. Reproduced with permission [87]. (**D**) Sol–gel transition and therapeutic effects of a thermosensitive hydrogel. Reproduced with permission [1].

Thus, the therapeutic value of temperature–responsive hydrogels depends not only on rapid in situ gelation but also on whether their post–gelation stability, mechanical adaptability, and degradation behavior are appropriately matched to the endometrial repair window. Figure 5 presents the functional properties of injectable, self-healing, adhesive, and thermosensitive hydrogels.

### 4.4. As a Physical Barrier: Prevention of Postoperative Re–Adhesion

#### 4.4.1. Spatial Occupancy and Mechanical Isolation

After hysteroscopic adhesiolysis, damage to the basal endometrial layer creates large exposed wound surfaces. During healing, abundant fibrin deposition and abnormal fibroblast proliferation can form fibrous bands that pull together the uterine walls, causing re–adhesion. After minimally invasive injection and in situ gelation, functional hydrogels can uniformly fill the uterine cavity and form a dense physical barrier between injured wound surfaces and the opposing uterine wall or adjacent tissues. This effectively prevents direct contact between wound tissues, blocks formation of fibrotic scar bridges, and reserves sufficient space for undisturbed endometrial regeneration and repair.

PEG–modified amino acid–based injectable hydrogels form stable physical barriers through hydrophobic–hydrophilic crosslinked networks and, without loading exogenous active substances, can effectively inhibit excessive fibroblast proliferation, reduce pathological fibrosis after uterine curettage, and create a favorable uterine environment for embryo implantation [38]. In addition, poly(N–(2–hydroxyethyl)acrylamide) (PHEAA) granular hydrogels, with excellent antifouling properties and stable network structures, can form a space–filling distribution after minimally invasive injection, establishing a reliable physical barrier in the uterine cavity. They inhibit abnormal protein and cell adhesion, promote endometrial regeneration, reduce fibrotic area, and significantly improve embryo implantation capacity in damaged endometrium [77]. Bioactive alginate hydrogels can also function as regenerative matrices in addition to providing physical support. For example, RGD–modified sodium alginate hydrogels carrying UCMSCs enhanced endometrial thickness and angiogenesis, reduced fibrosis, and improved pregnancy outcomes in an experimental endometrial injury model [65]. Physical isolation therefore represents a fundamental anti–adhesion function of hydrogel platforms and can provide a protected local environment for subsequent endometrial repair.

#### 4.4.2. Adaptability to Uterine Cavity Morphology

The uterine cavity is an irregular hollow structure that undergoes dynamic morphological changes with uterine peristalsis. Therefore, an effective intrauterine barrier should maintain close conformity to the endometrial surface during these changes without causing excessive mechanical compression or displacement. Compared with rigid or pre–shaped anti–adhesion devices, functional hydrogels provide greater structural flexibility and adaptability to the complex uterine cavity morphology.

Functional hydrogels constructed from CMC and oxidized HA combine injectability with intrinsic self–healing properties. Their flexible networks can conform to fine uterine folds and defects and maintain continuous coverage during morphological changes, thereby providing sustained physical separation of injured surfaces [98]. Thermosensitive poloxamer–based hydrogels similarly form flexible intrauterine barriers that can deform with uterine contractions, reducing the risks of displacement and local compression associated with rigid barrier materials [1]. In addition, decellularized amniotic membrane (dAM)–based thermosensitive hydrogels combine conformal coverage of irregular endometrial surfaces with retained natural matrix components, providing both physical protection and physiological support for endometrial regeneration [99]. Granular hydrogels further improve conformity to uterine folds and depressions through space–filling assembly, potentially overcoming the limited geometric adaptability of conventional bulk hydrogels [100].

Therefore, morphological adaptability is an important design requirement for maintaining continuous barrier coverage and effective physical isolation within the dynamically changing uterine cavity. A comparative overview of representative hydrogel classes is given in Table 3.

### 4.5. Intrinsic Antioxidant and Immunomodulatory Effects of Functional Hydrogels

Uterine surgical trauma induces local oxidative–stress imbalance and inflammatory cascades. Accumulated ROS damage normal endometrial cells and induce macrophage polarization toward the pro–inflammatory M1 phenotype, leading to secretion of large amounts of inflammatory factors and further aggravation of tissue injury and fibrosis. This is an important pathological driver of IUA development and progression. Through molecular structural design and component optimization, some functional hydrogels possess intrinsic antioxidant, anti–inflammatory, and immunomodulatory abilities and can actively remodel the pathological uterine microenvironment without loading drugs, cells, or other exogenous substances.

Hollow manganese dioxide nanozyme composite hydrogels possess intrinsic catalase– and superoxide dismutase–like activities and can directly and efficiently scavenge excessive ROS in the uterine cavity, relieve oxidative–stress injury to endometrial stromal cells, inhibit activation of oxidative–stress–mediated fibrotic signaling pathways, and regulate macrophage polarization balance to reduce local inflammation [23]. Self–healing adhesive functional hydrogels, such as P10G20, can capture free radicals through conjugated structures in their molecular chains, exhibit broad–spectrum antioxidant activity, effectively downregulate oxidative–stress levels in vivo, inhibit abnormal expression of the pro–fibrotic factor TGF–β1, and reduce collagen deposition and scar formation [33]. Hydrogel–based regenerative systems can also modulate the inflammatory microenvironment by suppressing pro–inflammatory cytokine expression and promoting macrophage polarization toward the reparative M2 phenotype. For example, hyaluronic acid hydrogels delivering mesenchymal stem cell–derived apoptotic bodies reduced the expression of inflammatory mediators such as TNF–α and IL–1β, enhanced M2 macrophage polarization, and consequently supported fibrosis reduction and functional endometrial regeneration [51]. In addition, oxidized dextran–gelatin adaptive membranes (GA–OD), owing to intrinsic antibacterial and anti–inflammatory properties, can inhibit intrauterine bacterial infection, regulate local inflammation, and promote epithelial reconstruction and cell proliferation, providing a clean and stable microenvironment for functional endometrial repair [75]. Dextran–lysozyme dynamic hydrogels likewise use intrinsic antibacterial and anti–inflammatory activity to regulate macrophage phenotype switching, inhibit pro–inflammatory factor release, and scavenge ROS, achieving coordinated anti–inflammatory, antioxidant, and anti–adhesion effects [95].

### 4.6. Intelligent Responsive Mechanisms and Programmable Design for IUA

The value of intelligent hydrogels lies not in simply stacking functions, but in matching material response logic with IUA pathological signals, uterine cavity structure, and therapeutic timing. For IUA, an ideal intelligent design should meet three requirements: first, achieve controlled activation within safe stimulation thresholds for the uterine cavity to avoid additional damage to the endometrium and embryo implantation potential; second, maintain structural stability during uterine contraction, uterine fluid flushing, and cyclic endometrial changes while permitting necessary repeated responses; and third, align drug–release sequence, tissue location, and stimulus response with the inflammatory, fibrotic, and regenerative stages.

Endogenous microenvironment–responsive systems represent one of the most disease–adapted intelligent strategies for IUA therapy. Their therapeutic value depends not only on responsiveness itself, but also on whether activation thresholds, response kinetics, and release duration are appropriately matched to the dynamic pathological changes during endometrial injury and repair. Compared with constant–rate release systems, programmed hydrogels may reduce unnecessary exposure of healthy endometrium while concentrating therapeutic signals at high–risk regions. Exogenous triggers, including ultrasound, magnetic fields, and light irradiation, further enable on–demand regulation of drug release or structural modulation; however, their clinical translation requires careful optimization of stimulus intensity, exposure duration, intrauterine localization, and reproductive safety to avoid additional injury to regenerating endometrium.

## 5. Therapeutic Cargo Delivery Strategies of Hydrogel Platforms for Endometrial Repair

Beyond serving as physical barriers, hydrogels provide versatile platforms for the localized delivery of therapeutic cargos to modulate the pathological microenvironment and promote functional endometrial regeneration. These strategies can be broadly categorized into the delivery of cell–free bioactive agents, including drugs, EVs or cell secretomes, growth factors, and PRP, and the delivery of living therapeutic cells, particularly stem cells. This section summarizes the major hydrogel–based delivery strategies and their applications in IUA therapy.

### 5.1. Drug–Loaded Hydrogels: Anti–Inflammatory, Anti–Fibrotic, and Pro–Regenerative Therapy

Small–molecule drugs, active components of traditional Chinese medicine, hormones, and related agents can directly regulate the injury microenvironment, suppress inflammation and fibrosis, and promote epithelial regeneration. However, systemic administration has significant side effects and makes it difficult to maintain local concentration. Hydrogels can provide uterine cavity–targeted, long–term sustained release while reducing toxicity and side effects.

Curcumin–loaded CMC–OHA hydrogels are representative systems for small–molecule anti–fibrotic delivery. Aldehyde groups in oxidized HA and amino groups in CMC form injectable, self–healing, degradable, and moderately tissue–adhesive dynamic networks through Schiff–base reactions. These networks gradually release curcumin, overcoming its poor water solubility, low stability, and insufficient bioavailability. Animal studies verify the favorable anti–fibrotic and fertility–improving effects of Cur@CMC–OHA hydrogels [105,106].

Estrogen (17beta–E2) is a core clinical drug for promoting structural endometrial repair. Thermosensitive heparin–poloxamer hydrogels loaded with E2 can provide long–term local sustained release, upregulate kisspeptin by activating ERK1/2 and p38 MAPK pathways, inhibit apoptosis, and promote endometrial regeneration [29,107]. Estradiol sustained–release hydrogels reflect a composite strategy of “barrier + local hormone therapy.” Tough PHEMA–based hydrogels can form stable networks through chemical crosslinking and Fe3+ coordination, and can combine estradiol–loaded silica for sustained release. In simulated uterine fluid, they maintain structural and mechanical stability for an extended period and continuously release estradiol to promote endometrial cell growth [30]. Such systems are suitable for discussing mechanical stability and long–term local delivery, but they also suggest that materials with excessive strength or prolonged stability require strict evaluation of uterine compliance, degradation or removal needs, and potential effects on the embryo implantation window.

In addition, multifunctional polysaccharide hydrogels can simultaneously scavenge ROS, suppress local inflammation and fibrosis, and promote endometrial regeneration, highlighting the value of microenvironment–responsive hydrogel platforms for integrated tissue repair [108]. Panax notoginseng saponin (PNS)–loaded CMC/OSA/PF127 double–network hydrogels can inhibit the NF–kappaB pathway and pro–inflammatory cytokine release while providing antibacterial, antioxidant, and pro–angiogenic synergy [98].

### 5.2. Exosome/Cell Secretome–Loaded Hydrogels for Cell–Free Therapy

Stem–cell–derived exosomes, EVs, and conditioned medium (CM) retain many of the paracrine bioactivities of their parent cells while offering lower immunogenicity, easier storage and quality control, and reduced tumorigenic concerns compared with living cell transplantation. However, free EVs and secreted factors are rapidly cleared and exhibit poor intrauterine retention, limiting their therapeutic duration. Hydrogel delivery systems can improve local retention, protect bioactive components, and provide sustained release within the uterine cavity.

Thermosensitive hydrogels loaded with human umbilical cord MSC–derived extracellular vesicles (hUCMSC–EVs) can significantly prolong EV retention and bioavailability in the uterine cavity and achieve better therapeutic outcomes than direct EV administration [4,32]. TSG6–modified exosomes incorporated into chitosan/β–glycerophosphate (CS/GP) thermosensitive hydrogels provide sustained local delivery and mitigate endometrial fibrotic damage [96]. Microfluidically prepared polydopamine–modified GelMA microsphere hydrogels loaded with VEGFA–overexpressing MSC exosomes combine EV delivery with tissue adhesion and ROS–scavenging capacity, promoting endometrial repair and improving fertility–related outcomes in IUA models [72]. Placental MSC conditioned medium (PC–MSC CM) combined with an adhesive hydrogel (CM/Gel–MA) forms a cell–free delivery system that supports endometrial stromal cell activity and tissue repair [54]. Decidual stromal cell–derived exosomes incorporated into alginate hydrogels provide another EV–delivery strategy for improving endometrial receptivity and fertility [109].

**Figure 6:**
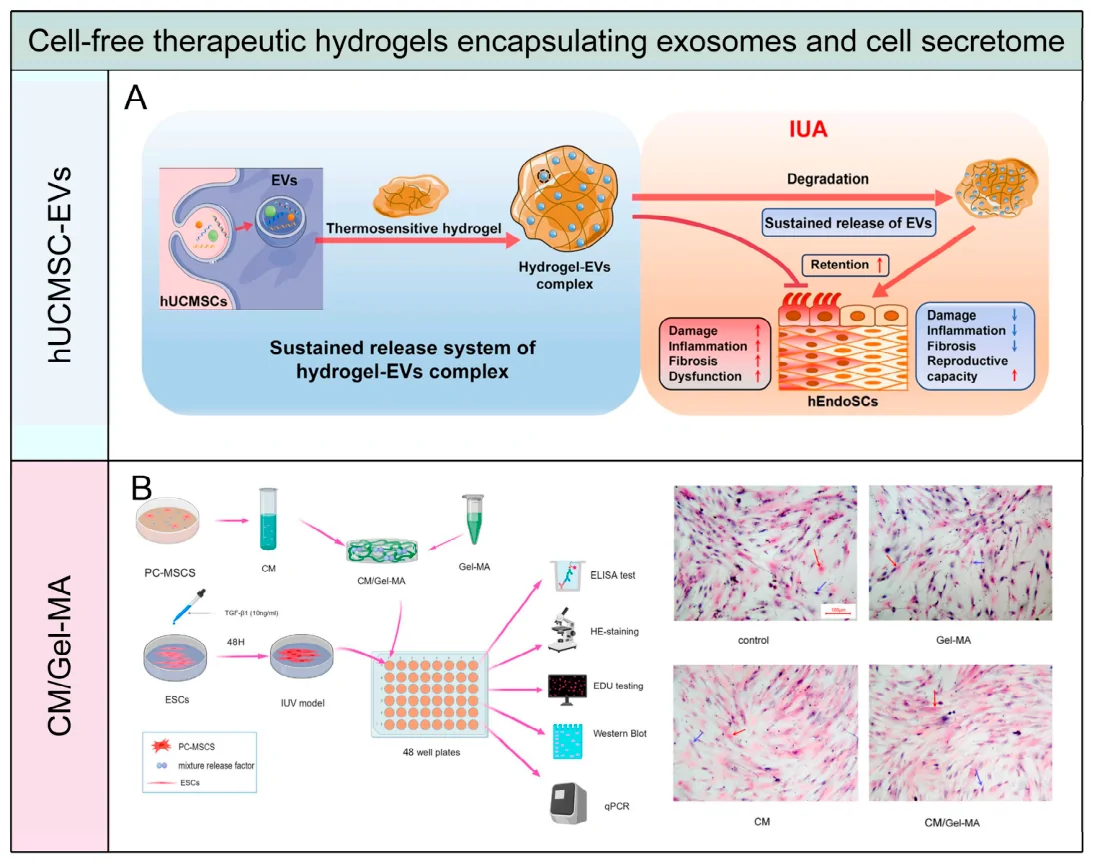
Hydrogel–mediated delivery of representative cell–free therapeutic cargos for endometrial regeneration. (**A**) Thermosensitive hydrogel–mediated delivery of hUCMSC–derived extracellular vesicles (hUCMSC–EVs) for sustained release and prolonged intrauterine retention. Reproduced with permission [32]. (**B**) Experimental design and anti–fibrotic evaluation of CM–loaded adhesive GelMA hydrogel. Reproduced with permission [54].

In addition to exosomes and other EVs, MSC–derived ABs can serve as cell–free regenerative signal carriers containing functional molecules such as miRNAs, mRNAs, proteins, and lipids [4]. AB–loaded HA hydrogels provide sustained intrauterine retention and release of these regenerative signals, promoting endometrial repair and fertility restoration in mouse acute endometrial injury and rat IUA models [51]. This strategy expands hydrogel–mediated cell–free therapy beyond the conventional exosome–delivery paradigm. Hydrogel-mediated delivery of exosomes and conditioned medium is depicted in Figure 6.

### 5.3. Growth Factor– and PRP–Loaded Hydrogels

Growth factors such as VEGF, bFGF, FGF1, and KGF, as well as PRP, are rich in pro–regenerative active factors and can strongly promote angiogenesis, cell proliferation, and glandular reconstruction. However, direct application is limited by burst release, easy inactivation, and poor retention. Hydrogels can enable stable encapsulation, controlled release, and localized activation of growth factors, improving repair efficiency. PRP–loaded hydrogels are attractive because they provide a mixture of pro–angiogenic and pro–regenerative cues; however, PRP composition varies markedly among patients, and standardized activation, loading, and release protocols are required before reproducible clinical application.

Genetically engineered FGF1–sericin hydrogels enable stable growth factor release, effectively restore endometrial morphology, and achieve a high fertility rate of 65.1% in rat models [15]. A dual growth–factor sequential delivery system releases VEGF first to promote angiogenesis and IGF–1 later to support tissue remodeling, significantly improving embryo implantation and live–birth rates [29]. Dynamic phenylboronic ester double–network hydrogels can achieve localized PRP activation and long–term growth–factor release, inhibit the TGF–β1/SMAD2/3 pathway, reduce collagen deposition, and promote endometrial regeneration and functional recovery [38]. L–serine–induced alginate hydrogels loaded with PRP can rapidly gel, adapt to uterine cavity mechanical features, release PRP growth factors for an extended period, reduce collagen deposition, and promote endometrial cell proliferation [92]. Heparin–poloxamer hydrogels loaded with keratinocyte growth factor (KGF) can significantly improve mucosal adhesion and drug absorption and promote epithelial cell, glandular, and vascular proliferation [87]. Autologous PRP double–network hydrogels can activate the WNT pathway, upregulate SOX9/LGR5, and promote human endometrial gland regeneration and clinical repair [110].

PRP–delivery hydrogels are also evolving from “simple encapsulation” toward “local activation and controlled release.” Chitosan–lignin/poloxamer (CL–PF127@PRP) hydrogels use electrostatic interactions between chitosan amino groups and lignosulfonate groups to strengthen the PF127 thermosensitive network and improve PRP retention and release. This system has good injectability, appropriate degradation, and PRP release behavior, shows anti–inflammatory and pro–angiogenic activity in vitro, and increases endometrial thickness and receptivity in animal experiments [111]. Together with dynamic boronate ester double–network hydrogels for local PRP activation, such studies indicate that PRP hydrogels should focus on optimizing the activation timing, release profile, and uniform uterine coverage of growth factors [38].

### 5.4. Stem–Cell–Laden Hydrogels for Living Cell Delivery

Unlike drugs, growth factors, and EVs, stem cells are living therapeutic entities that require a supportive microenvironment rather than simple encapsulation and release. Hydrogel–based stem cell delivery systems can provide a three–dimensional biomimetic niche that improves cell retention, survival, engraftment, and paracrine activity within the injured uterine cavity. MSCs, endometrial stem/progenitor cells, and related therapeutic cells can contribute to endometrial repair through immunomodulatory, pro–angiogenic, anti–fibrotic, and paracrine effects. Therefore, hydrogel design for stem cell therapy should focus not only on efficient cell delivery but also on maintaining cell viability and regenerative function within the pathological intrauterine microenvironment.

Several hydrogel platforms have been developed to overcome the limitations of direct stem cell transplantation, including poor survival, insufficient retention, and reduced therapeutic activity in the inflammatory and oxidative intrauterine microenvironment. Injectable hydrogels constructed through Diels–Alder click chemistry and loaded with human umbilical cord mesenchymal stem cells (UCMSCs) provide suitable mechanical properties and biocompatibility, enabling minimally invasive delivery and adaptation to irregular uterine cavity morphology. In rat IUA models, these hydrogels promoted M2 macrophage polarization, reduced fibrosis, enhanced angiogenesis, and improved endometrial repair and fertility restoration [23].

Beyond simple cell encapsulation, advanced hydrogel systems aim to regulate the stem cell niche and maintain cellular function. GelMA/ColMA composite hydrogels fabricated by 3D bioprinting and loaded with amniotic mesenchymal stem cells (AMSCs) possess interconnected microporous structures and stable mechanical properties, supporting sustained cell retention and endometrial structural reconstruction in rat IUA models [60]. Thermosensitive hydrogels incorporating adipose–derived mesenchymal stem cells (ADSCs) and melanin nanoparticles further enhance the local regenerative microenvironment by promoting expression of bFGF, IGF–1, and VEGFA, thereby improving vascular regeneration, endometrial thickness, and receptivity [97].

Hydrogels can also be combined with clinically relevant biomaterials to improve translational potential. Clinical–grade fibrin hydrogels loaded with UCMSCs promote endometrial repair through combined immune regulation and tissue regeneration, while a cell–free fibrin hydrogel–derived EVs system achieves comparable therapeutic effects, suggesting the potential of both cellular and cell–free strategies [112]. In addition, RGD–modified alginate hydrogels and chitosan–based thermosensitive hydrogels enhance stem cell adhesion, engraftment, and paracrine function, leading to improved therapeutic outcomes in IUA models [64,88].

**Figure 7:**
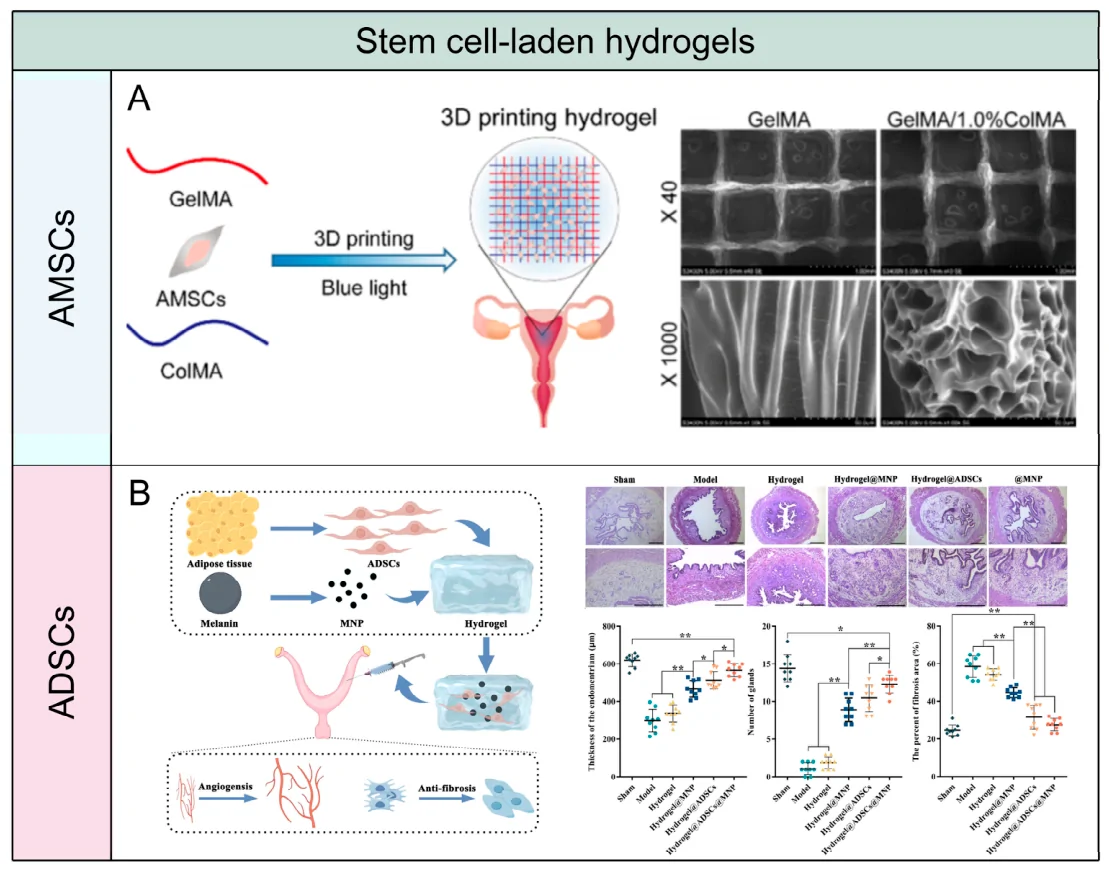
Hydrogel–mediated delivery of stem cells for IUA therapy and endometrial regeneration. (**A**) 3D–bioprinted GelMA/ColMA composite hydrogel encapsulating AMSCs, with characterization of its microporous scaffold structure. Reproduced with permission [60]. (**B**) Thermosensitive hydrogel co–delivering ADSCs and melanin nanoparticles, showing the proposed therapeutic mechanism and in vivo endometrial repair outcomes. * indicates *p* < 0.05, and ** indicates *p* < 0.01. Reproduced with permission [97].

A further trend in stem–cell–laden hydrogel design is the transition from improving cell retention toward actively regulating the transplanted cell microenvironment. TPG antioxidant hydrogels containing E7 peptide–modified gelatin microspheres provide ROS–scavenging and self–healing properties, protect UCMSC activity under oxidative stress, and promote M2 macrophage polarization, thereby enhancing endometrial regeneration and fertility recovery. Figure 7 illustrates the design and outcomes of stem-cell-laden hydrogel systems. However, despite encouraging preclinical results, clinical translation of stem–cell–laden hydrogels remains limited by challenges related to cell source variability, manufacturing standardization, long–term safety, immune compatibility, and reproductive outcomes.

### 5.5. Controlled–Release Mechanisms and Kinetics of Hydrogel Delivery Systems

Hydrogel–mediated therapeutic delivery is regulated by the interplay among cargo diffusion, polymer degradation, swelling, network relaxation, and cargo–matrix interactions. These processes can be adjusted through crosslinking density, mesh size, dynamic chemical bonds, and stimulus–responsive structures. Thermosensitive hydrogels enable minimally invasive injection and in situ gelation for improved intrauterine retention [1,96], while pH– and ROS–responsive systems achieve microenvironment–responsive release through changes in network structure or degradation behavior [113]. Dynamic covalent bonds, including Schiff–base and phenylboronic ester linkages, further provide reversible remodeling and controlled release properties [23,38]. Double–network hydrogels and microsphere/microcapsule–integrated systems can optimize diffusion pathways and achieve sustained or sequential delivery [98,110].

Quantitative kinetic analysis provides a useful means of identifying dominant release mechanisms and optimizing hydrogel formulations. Experimental release profiles are commonly fitted using zero–order, first–order, Higuchi, and Korsmeyer–Peppas models [114,115]. In particular, the release exponent derived from the Korsmeyer–Peppas model can help distinguish diffusion–dominated, polymer–relaxation–controlled, and anomalous transport, while the Peppas–Sahlin model can further estimate the relative contributions of diffusion and polymer relaxation [115]. Such analyses allow release behavior to be related to hydrogel properties rather than being described solely by cumulative release percentages.

Beyond empirical curve fitting, recent model–based approaches integrate experimentally determined parameters, including diffusivity, swelling, degradation, hydrogel geometry, and cargo distribution, to predict release profiles and guide material design [114,116]. Computational simulations can further evaluate how changes in crosslinking density, network structure, carrier dimensions, and initial cargo distribution influence release kinetics before extensive experimental optimization [116,117]. Statistical approaches such as response–surface modeling have also been used to establish quantitative relationships between formulation variables and drug–release behavior, enabling prediction of release profiles within defined design spaces [118].

Therefore, hydrogel delivery design is increasingly shifting from empirical formulation toward mechanism–guided and predictive optimization. Integrating experimental release testing with kinetic fitting and computational modeling can help identify key transport parameters and guide rational adjustment of hydrogel structure to achieve cargo–specific release profiles. For IUA therapy, such approaches may facilitate more precise coordination between hydrogel degradation, therapeutic delivery, and endometrial regeneration.

Overall, current evidence does not establish a clear therapeutic hierarchy between cellular and acellular hydrogel delivery systems. Stem–cell–laden hydrogels may provide sustained paracrine and immunomodulatory activity but face greater challenges in cell sourcing, manufacturing, storage, immune compatibility, and reproductive safety. In contrast, acellular systems generally offer greater controllability and translational feasibility, although their efficacy depends strongly on cargo stability and release kinetics. Notably, clinical–grade fibrin hydrogels delivering UCMSCs or their EVs have shown comparable therapeutic effects in preclinical endometrial injury models [112]. Future studies should therefore directly compare these strategies in terms of regenerative efficacy, reproductive outcomes, safety, manufacturing reproducibility, and scalability rather than assuming that greater biological complexity confers superior therapeutic benefit.

## 6. Mechanistic Evidence and Therapeutic Endpoints of Hydrogel–Based IUA Therapy

**Figure 8:**
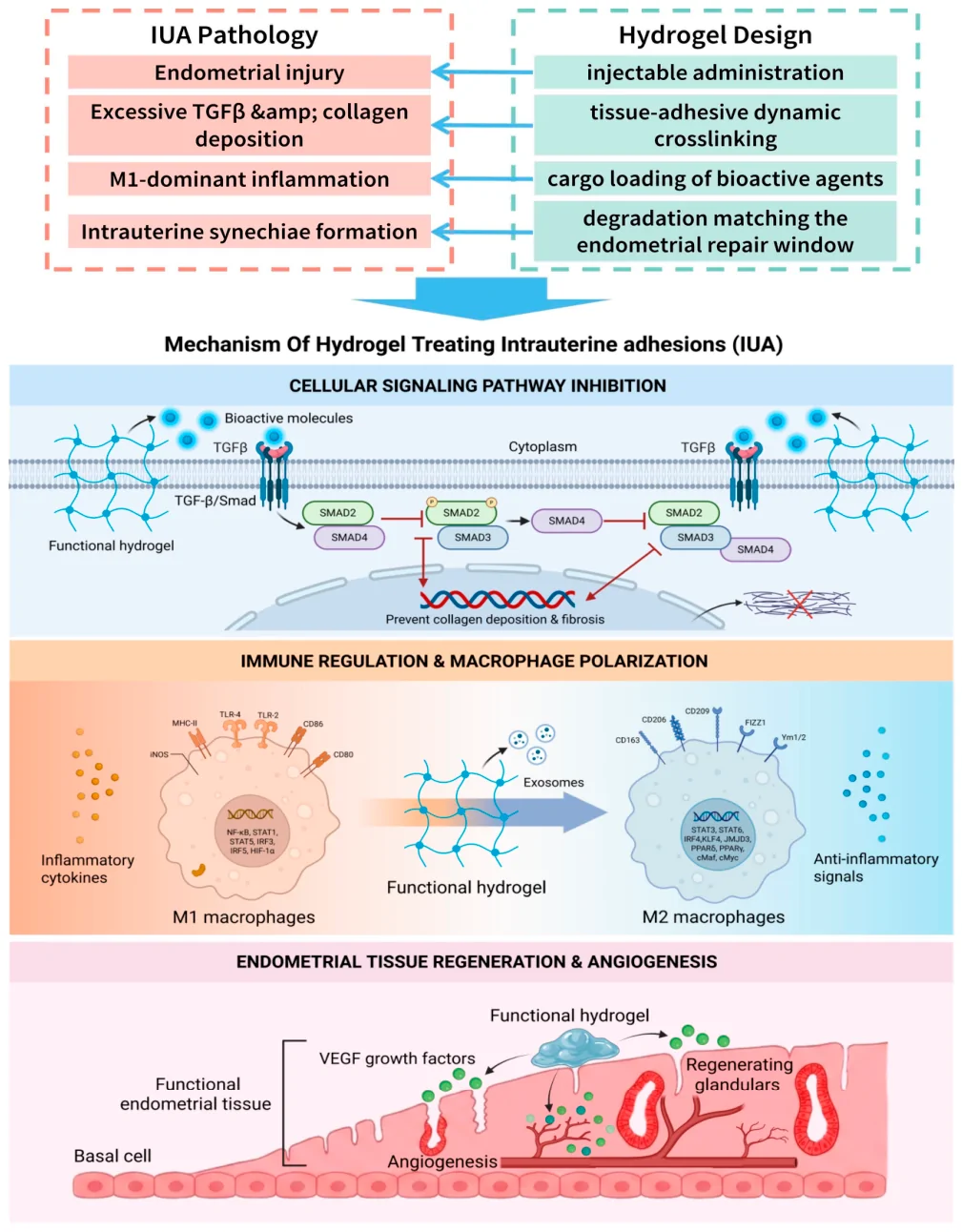
Schematic of IUA pathology–guided hydrogel design and its therapeutic mechanisms for endometrial repair. Created with BioRender.com.

Hydrogel–based IUA therapies exert therapeutic effects through interconnected anti–fibrotic, immunomodulatory, and pro–regenerative mechanisms. Beyond providing physical separation of injured surfaces, functional hydrogels can modulate the pathological microenvironment and promote coordinated recovery of endometrial structure and function. This section focuses on three major therapeutic mechanisms: inhibition of fibrosis, regulation of the immune microenvironment, and promotion of angiogenesis and endometrial regeneration, together with the corresponding evidence of functional recovery (Figure 8).

### 6.1. Inhibition of Fibrosis and Regulation of the TGF–β/Smad Signaling Pathway

As discussed in Section 2.2, TGF–β1/Smad signaling is a central fibrotic axis in IUA and represents a major molecular target of hydrogel–based interventions. Therapeutic suppression of this pathway can reduce EMT, myofibroblast activation, and excessive ECM deposition, thereby limiting endometrial fibrosis [106].

Numerous studies have confirmed that functional hydrogels can inhibit this pathway through multidimensional loading of drugs, cytokines, or bioactive components to reverse fibrosis. Curcumin–loaded injectable hydrogels can significantly downregulate TGF–β1 expression, inhibit EMT, reduce fibrotic tissue formation, and thereby prevent IUA [5,105]. Genetically engineered FGF1–sericin hydrogels can stably release FGF1, directly inhibit activation of the TGF–β/Smad pathway, and block transition of endometrial stromal cells toward a fibrotic phenotype [15]. PRP–loaded double–network hydrogels inhibit TGF–β1–Smad2/3 phosphorylation, downregulate fibrotic markers such as Collagen I and α–SMA, and reduce abnormal ECM deposition [38,110]. Poly(ethylene glycol)–b–poly(L–phenylalanine)/poly(ethylene glycol) hydrogels can directly inhibit uterine cavity fibrosis by regulating the interaction between TGF–β1 and Muc–4 [26].Together, these results indicate that targeted inhibition of the TGF–β/Smad pathway and its upstream and downstream inflammation–oxidative stress axis is a core molecular mechanism of hydrogels in IUA treatment [3,80,119].

### 6.2. Regulation of the Immune Microenvironment and Macrophage Polarization

Macrophage polarization is an important therapeutic target in IUA, and hydrogel–based interventions can modulate macrophage dynamics through intrinsic bioactivity or the delivery of stem cells, exosomes, ABs, and other immunomodulatory factors. By suppressing excessive inflammatory responses and promoting a repair–supportive immune microenvironment, these strategies can interrupt the inflammation–fibrosis cascade and facilitate endometrial regeneration [20,24].

HA hydrogels delivering stem–cell–derived ABs can regulate macrophage function, reduce local inflammation, and promote repair [51]. Engineered EVs loaded with STAT1–siRNA and delivered by adhesive hydrogels can inhibit STAT1 phosphorylation, reduce generation of M1 macrophages, and thereby decrease myofibroblast activation and collagen deposition [39]. TSG6–modified exosomes combined with thermosensitive hydrogels can directly inhibit excessive macrophage activation, balance the M1/M2 ratio, and alleviate endometrial fibrosis [25,96].

At the same time, self–healing adhesive hydrogels can reduce in vivo oxidative stress, downregulate TGF–β1 and inflammatory factors, and promote reparative macrophage polarization through combined antioxidant and immunomodulatory effects [33,76]. Decidualized matrix hydrogels can synchronously reprogram macrophage phenotype, inhibit pyroptosis and TGF–β/Smad3 signaling, and achieve dual immune–fibrotic regulation [17]. Mussel–inspired adhesive HMD hydrogels can prolong stem–cell retention, activate the PI3K/Akt pathway, increase the M2/M1 ratio, and promote restoration of the immune microenvironment [48]. These studies confirm that regulating macrophage polarization and suppressing excessive inflammation are key immune mechanisms by which hydrogels prevent adhesion formation [32,120]. Because excessive M2 polarization may also contribute to fibrotic remodeling under certain conditions, future hydrogel studies should evaluate macrophage dynamics over time rather than using a single M1/M2 ratio at one endpoint.

### 6.3. Promotion of Angiogenesis and Functional Endometrial Regeneration

Adequate angiogenesis is the foundation for endometrial structural reconstruction, glandular regeneration, and functional recovery, as it improves local blood supply and supports cell proliferation and tissue remodeling [41,46]. Hydrogels drive endometrial regeneration by loading pro–angiogenic factors, cell secretomes, stem cells, or biomimetic matrices.

A dual growth–factor sequential delivery system (VEGF + IGF–1) can mimic the physiological regeneration sequence, with VEGF first initiating angiogenesis and IGF–1 subsequently promoting tissue remodeling, significantly improving recovery of endometrial structure and function [29,121]. PRP–loaded double–network hydrogels upregulate VEGFA expression, activate the PI3K/AKT pathway, and strongly promote angiogenesis and endometrial cell proliferation [122,123]. Sprayable peptide amphiphile hydrogels loaded with human endometrial organoid extracellular vesicles (HEO–EVs) can promote angiogenesis and inhibit local fibrosis [124].

Heparin–poloxamer hydrogels loaded with KGF significantly promote angiogenesis, epithelial proliferation, and glandular regeneration [87]. Thermosensitive hydrogels combining ADSCs and melanin upregulate bFGF, IGF–1, and VEGFA, accelerate vascular and glandular regeneration, and restore endometrial receptivity [97]. Alginate hydrogels combined with super–active platelet lysate (sPL) prolong cytokine action, promote CD34+ angiogenesis, and repair the endometrium [125]. 3D–printed porous scaffolds loaded with stem cells promote vascularization and functional endometrial regeneration by improving the local microenvironment [60,86].

In addition, recombinant type III collagen–hyaluronic acid biomimetic hydrogels promote vascular and glandular reconstruction and restore fertility by enhancing cell adhesion and proliferation [1,59]. Endometrial decellularized matrix hydrogel (EndoGel) mimics the natural microenvironment and significantly promotes angiogenesis and functional regeneration [124,126]. These findings indicate that angiogenesis–centered endometrial regeneration is the final effector pathway through which hydrogels restore uterine fertility [62,63]. An increase in endometrial thickness alone should not be interpreted as functional endometrial regeneration unless accompanied by glandular reconstruction, vascular perfusion, receptivity markers, and fertility–related outcomes.

## 7. Translational Roadmap for Hydrogel–Based IUA Therapy

Although most hydrogel–based IUA therapies remain at the preclinical stage, several systems have shown early translational promise and require standardized validation before clinical adoption. Some gels based on mature materials have entered clinical exploration. For example, combining autologous PRP with double–network (DN) hydrogels has been used clinically in patients with moderate–to–severe IUA. The results showed that this hydrogel has been reported to improve menstrual volume, subendometrial blood flow, IUA scores, and pregnancy–related outcomes in preliminary clinical exploration; however, larger randomized and controlled studies are still required [110]. This provides direct evidence for clinical hydrogel application. In addition, injectable granular zwitterionic poly(sulfobetaine methacrylate) (G–PSBMA) hydrogels, owing to antifouling properties that reduce non–specific adhesion, good biocompatibility, and adaptability to uterine contours, inhibit fibrosis and inflammation and restore fertility in animal models, showing promising clinical translational prospects [127]. The key translational requirements are summarized in Table 4.

## 8. Future Perspectives

### 8.1. Technical Challenges

Optimization of hydrogel material properties is the primary technical challenge for clinical translation. First, matching degradation rate with tissue regeneration rate is crucial. An ideal anti–adhesion hydrogel should provide an effective physical barrier during the critical period of endometrial repair, usually 1–2 weeks, and then gradually degrade and be absorbed by the body to avoid secondary surgical removal. However, degradation rates vary markedly among hydrogels; for example, HA–based hydrogels degrade relatively quickly, whereas PEG– or PHEMA–based hydrogels may degrade too slowly and may even require surgical removal [30]. Second, mechanical strength must match the dynamic uterine cavity environment. Hydrogels require sufficient mechanical performance to resist uterine peristalsis and intrauterine pressure and prevent compression or expulsion during healing. For example, introducing double–network structures, such as calcium alginate/polyacrylamide, or covalent crosslinking can significantly improve elastic modulus and stretchability [68]. However, excessive mechanical strength may make the material stiff, preventing it from conforming to the irregular uterine cavity and impairing barrier function. In addition, the choice of sterilization method directly affects material performance. Conventional moist–heat sterilization or ethylene oxide sterilization may damage the chemical structure of hydrogels or bioactive factors such as growth factors and EVs, whereas irradiation sterilization may cause polymer chain scission or crosslinking. Therefore, developing a standardized process that provides effective sterilization without impairing material function is an obstacle that must be overcome as hydrogels move from laboratory research to clinical application.

### 8.2. Biological Challenges

The in vivo biological behavior of hydrogels is critical for successful application. The primary challenge is host immune response. After implantation, hydrogels trigger a series of immune responses, including infiltration by macrophages, neutrophils, and other immune cells. Although moderate inflammation initiates tissue repair, excessive and persistent inflammation aggravates fibrosis and opposes therapeutic goals. Studies suggest that regulating macrophage polarization, for example by promoting the M2 anti–inflammatory phenotype, is an effective strategy to reduce immune responses. MSC–loaded hydrogels can induce M2 macrophage polarization through paracrine activity and thereby inhibit fibrosis [23]. However, hydrogels themselves or their degradation products may also act as foreign bodies and induce chronic inflammation or granuloma formation. Second, long–term safety data are lacking. Most current studies are limited to short–term observations, from weeks to months, in small animal models such as rats, and lack safety assessments in large animal models such as non–human primates and over longer periods, such as more than one year. The in vivo metabolic pathways, potential cytotoxicity, and carcinogenicity of hydrogel degradation products remain unclear. Finally, potential effects on fertility are an unavoidable core issue in clinical translation. Although many hydrogels are designed to promote endometrial regeneration and restore fertility [15], whether the material itself or its degradation products affect oocyte quality, embryo implantation, or early embryonic development requires rigorous reproductive toxicology evaluation. For example, some crosslinkers or unreacted monomers may have reproductive toxicity; therefore, selecting natural polymers with excellent biocompatibility and non–toxic degradation products, such as HA and chitosan, or synthetic polymers such as PEG, is key to reducing risk [29].

### 8.3. Progress and Prospects in Integrating Intelligent Gel Materials with Digital Technologies

Digital technologies are providing a closed–loop foundation for intelligent hydrogel therapy in IUA, spanning diagnosis, delivery, and efficacy evaluation. Preoperatively, AI–assisted hysteroscopic image recognition, three–dimensional ultrasound, or MRI reconstruction can be used to assess adhesion extent, uterine cavity morphology, endometrial thickness, blood–flow perfusion, and fibrosis degree, thereby supporting patient stratification and material selection. For example, patients with mild–to–moderate disease may preferentially receive antifouling, injectable, degradable barrier gels, whereas severe or recurrent patients may require composite hydrogels delivering exosomes, PRP, nucleic acids, or growth factors.

Intraoperative digital delivery technologies can improve the operability of materials within the uterine cavity. Hysteroscopy–guided visualized injection, spraying devices, and navigation delivery systems are expected to improve coverage uniformity and localization accuracy on irregular wound surfaces, reducing material accumulation, omission, or loss. For injectable in situ gelation systems, digital imaging can also be used to evaluate uterine cavity filling range, gel retention position, and early postoperative degradation, extending material performance assessment from simple in vitro metrics to real anatomical scenarios.

At the research and validation levels, 3D printing, microfluidics, organoids, and endometrium–on–chip technologies can help establish preclinical models that more closely resemble the human uterine cavity microenvironment. 3D printing can build personalized scaffolds, gradient gels, and spatially partitioned materials according to uterine cavity morphology or lesion distribution. Microfluidic platforms can simulate uterine fluid flushing, inflammatory gradients, and hormonal–cycle changes. Endometrial organoid and chip models can evaluate the effects of hydrogels on re–epithelialization, vascularization, gland formation, and endometrial receptivity. In the future, if further integrated with soft sensors, bioelectronic interfaces, and responsive gels, short–term monitoring of local pH, ROS, temperature, inflammatory factors, or mechanical state together with on–demand release will become an important conceptual basis for closed–loop uterine repair.

### 8.4. Future Directions: Pathology Orientation, Stage Matching, and Intelligent Translation

Overall, hydrogel–based strategies provide promising approaches for IUA repair. Rational material design should balance structure–function properties, pathological staging requirements, reproductive safety and clinical manufacturability. Although numerous pre–clinical achievements have been obtained, multiple technical and biological barriers remain before widespread clinical adoption. Future efforts should move beyond material–oriented innovation toward pathology–guided, patient–stratified, and translation–oriented hydrogel development for endometrial regeneration.

Looking ahead, the core trend in IUA hydrogel therapy is a shift from “material–driven” to “pathology–driven” design. In other words, material design should first answer which pathological stage the patient is in, what therapeutic signals are needed in the injured region, and what mechanical and spatial adaptation the uterine cavity requires, rather than simply pursuing more functions. Mild or postoperative–prevention IUA requires safe, degradable, antifouling, and morphology–adaptive barrier materials. Moderate IUA requires responsive hydrogels with anti–inflammatory, antioxidant, and anti–fibrotic capacity. Severe or recurrent IUA may require combined strategies such as ECM biomimicry, exosome/apoptotic body delivery, stem cells, PRP, growth factors, or gene therapy delivery to move from morphological restoration to functional endometrial reconstruction.

Currently, the most translationally promising directions are injectable, in situ–gelling, degradable intelligent hydrogels with relatively clear manufacturing processes, including thermosensitive hydrogels, HA/chitosan dynamic crosslinked hydrogels, antifouling granular hydrogels, and PRP or exosome sustained–release hydrogels. These systems should prioritize solving uterine cavity retention, coverage uniformity, degradation window, sterilization method, and batch consistency, and should simultaneously evaluate re–adhesion rate, endometrial thickness, gland number, blood flow, fibrotic area, and pregnancy outcomes in preclinical models. At this stage, the focus is not on proposing complex concepts, but on establishing standardized animal models, unified efficacy endpoints, and reproducible material quality–control systems.

Overall, developing intelligent gels according to pathology, disease characteristics, tissue structure, and disease–course dynamics, and using digital technologies for design, delivery, and validation, will be important for improving IUA treatment and promoting interdisciplinary development. The integration of intelligent gels and digital technologies is expected to move IUA therapy gradually toward precision, minimal invasiveness, monitorability, and personalization.

## 9. Conclusions

In summary, hydrogel–based platforms for IUA therapy have evolved from passive anti–adhesion barriers into multifunctional systems capable of local delivery, microenvironmental regulation, anti–fibrotic intervention, immunomodulation, and endometrial regeneration. Their therapeutic value depends not only on the choice of polymer backbone but also on network architecture, gelation mode, wet adhesion, degradation kinetics, and stage–matched release behavior.

Future development should shift from function accumulation to disease–specific and translation–oriented design. Clinically useful IUA hydrogels should be injectable, conformable, reproductive–safe, residue–free after the repair window, and compatible with standardized manufacturing and intrauterine delivery. More rigorous validation using clinically relevant models, fertility–related endpoints, and long–term safety assessment will be essential for moving hydrogel–based IUA therapy from preclinical promise to clinical application.

## Figures and Tables

**Table 1:** Pathological cues in IUA and corresponding hydrogel design strategies.

Pathological Cue	Main Biological Consequence	Hydrogel Design Requirement	Possible Material Strategy
ROS accumulation	Apoptosis, inflammation, fibrosis	Antioxidant microenvironment regulation	Polyphenol, nanozyme, ROS–responsive bonds
TGF–β/Smad activation	EMT, myofibroblast activation	Sustained anti–fibrotic release	Curcumin, aspirin, siRNA, growth factor modulation
M1 macrophage dominance	Inflammatory cascade	Immunomodulatory delivery	EVs, ABs, cytokine–regulating hydrogels
Poor angiogenesis	Thin endometrium, low receptivity	Pro–vascular regeneration	VEGF, bFGF, PRP, exosome delivery
Irregular uterine cavity	Poor retention	Wet adhesion and self–healing	Catechol, Schiff base, dynamic covalent network

**Table 2:** Polymeric materials used in hydrogel system for IUA treatment.

Material Type	Representative Material	Core Advantages	Main Limitations	Typical Improvement Strategies	Ref.
Natural polymer	HA	Excellent biocompatibility; participates in tissue repair signaling	Weak mechanical properties; rapid degradation	Chemical crosslinking; composite formation with gelatin	[48]
Natural polymer	Chitosan (CS)	Intrinsic antibacterial/hemostatic activity; promotes cell adhesion	Low solubility at neutral pH	Chemical modification (ACS); thermosensitive composites	[49]
Natural polymer	Gelatin/GelMA	Contains RGD cell–adhesion motifs; controllable photocrosslinking	Thermally unstable; limited mechanical properties	Methacrylation; composite formation with PF–127	[50]
Natural polymer	Alginate (SA)	Mild gelation conditions; good biodegradability	Lack of cell–adhesion sites	Composite formation with CS/PEG	[51]
Synthetic polymer	PEG/PHEMA	Precisely tunable mechanical properties; low immunogenicity	Lack of bioactivity; requires functional modification	Grafting of RGD sequences; loading of bioactive factors	[52]
Synthetic polymer	Poloxamer (PF–127)	Thermosensitive in situ gelation; excellent injectability	Low mechanical strength; rapid degradation	Composite formation with GelMA/collagen	[53]
Composite/Hybrid	Multicomponent composite system	Multifunctional synergy and complementary properties	Complex preparation and difficult quality control	Fine regulation of component ratios and crosslinking density	[54]

**Table 3:** Comparative characteristics of representative hydrogel classes relevant to IUA therapy.

Hydrogel Class	Biocompatibility	Wet–Tissue Adhesion	Injectability	Degradation Tunability	Drug–Delivery Capacity	Manufacturing Reproducibility	Ref.
Natural–derived (HA, gelatin, alginate)	High	Moderate	Good	Moderate–high	Moderate–high	Moderate	[101]
Synthetic hydrogels (Pluronic–based)	Good	Low–moderate	Excellent	Low–moderate	High	High	[101,102]
Dynamic self–healing hydrogels	Good–high	Moderate–high	Excellent	High	High	Moderate	[103]
Stimulus–responsive hydrogels	Good	Variable	Moderate–excellent	High	Very high	Moderate–low	[104]

**Table 4:** Key translational requirements for hydrogel–based IUA therapies.

Translational Issue	Main Risk	Required Evaluation	Suggested Solution
Sterilization	network damage, cargo inactivation	rheology, degradation, release profile before/after sterilization	aseptic preparation, filtration, mild sterilization
Batch consistency	unstable gelation and release	polymer MW, substitution degree, viscosity, G′/G″	GMP–grade raw materials
Residual crosslinker	cytotoxicity, reproductive toxicity	LC–MS, cytotoxicity, implantation assay	low–toxicity dynamic bonds
Uterine retention	rapid loss by fluid/peristalsis	imaging–based retention assay	wet adhesive/self–healing design
Mechanical mismatch	compression injury or early collapse	rheology and fatigue tests	soft but stable network
Reproductive safety	implantation failure, fetal risk	pregnancy rate, live birth, offspring follow–up	degradable and residue–free design
Clinical endpoint	weak clinical relevance	AFS score, menstrual recovery, pregnancy/live birth	standardized endpoint panel

## Data Availability

No new data were created or analyzed in this study. Data sharing is not applicable to this article.

## Abbreviations

ABs	Apoptotic bodies
ADSCs	Adipose–derived stem cells
AMSCs	Amniotic mesenchymal stem cells
bFGF	Basic fibroblast growth factor
CASH	Colloid–assembled self–healing
CM	Conditioned medium
CMC	Carboxymethyl chitosan
CS	Chitosan
ECM	Extracellular matrix
EMT	Epithelial–mesenchymal transition
EVs	Extracellular vesicles
GelMA	Gelatin methacryloyl
HA	Hyaluronic acid
hUCMSC–EVs	Human umbilical cord mesenchymal stem cell–derived extracellular vesicles
IUA	Intrauterine adhesion
KGF	Keratinocyte growth factor
MSC	Mesenchymal stem cell
NIR	Near–infrared
PEG	Polyethylene glycol
PHEMA	Poly(2–hydroxyethyl methacrylate)
PNS	Panax notoginseng saponins
PRP	Platelet–rich plasma
PVA	Polyvinyl alcohol
ROS	Reactive oxygen species
SA	Sodium alginate
TCRA	Transcervical resection of adhesions
TGF–β1	Transforming growth factor–beta 1
UCMSCs	Umbilical cord mesenchymal stem cells
VDR	Vitamin D receptor
